# Transient structures in rupturing thin films: Marangoni-induced symmetry-breaking pattern formation in viscous fluids

**DOI:** 10.1126/sciadv.abb0597

**Published:** 2020-07-08

**Authors:** Li Shen, Fabian Denner, Neal Morgan, Berend van Wachem, Daniele Dini

**Affiliations:** 1 Department of Mechanical Engineering, Imperial College London, Exhibition Road, London SW7 2AZ, UK.; 2 Chair of Mechanical Process Engineering, Otto-von-Guericke-Universität Magdeburg, Universitätsplatz 2, 39106 Magdeburg, Germany.; 3 Shell Global Solutions Ltd., Shell Centre York Road, London SE1 7NA, UK.

## Abstract

In the minutes immediately preceding the rupture of a soap bubble, distinctive and repeatable patterns can be observed. These quasistable transient structures are associated with the instabilities of the complex Marangoni flows on the curved thin film in the presence of a surfactant solution. Here, we report a generalized Cahn-Hilliard-Swift-Hohenberg model derived using asymptotic theory that describes the quasielastic wrinkling pattern formation and the consequent coarsening dynamics in a curved surfactant-laden thin film. By testing the theory against experiments on soap bubbles, we find quantitative agreement with the analytical predictions of the nucleation and the early coarsening phases associated with the patterns. Our findings provide fundamental physical understanding that can be used to (de-)stabilize thin films in the presence of surfactants and have important implications for both natural and industrial contexts, such as the production of thin coating films, foams, emulsions, and sprays.

## Teaser

A transient pattern formation on the top of a soap bubble induced by surface tension gradients is examined via asymptotics.

## INTRODUCTION

The Marangoni effect inherently produces nonlinear structures within fluid flow; the formation of these structures will inevitably lead to symmetry breaking within the system. The premise of symmetry breaking is present in a wide range of phenomena, not least in the interfacial fluid context, from the Higgs mechanism (*1*) in particle physics to solid crystallization (*2*) and to the functions of the cell structure (*3*). The analysis of these symmetry-breaking phenomena typically involves a reduction of the complex system into field variables, which then form an effective field theory. The Ginzburg-Landau theory of phase transitions (*4*), the Cahn-Hilliard theory of phase separation (*5*), the Swift-Hohenberg theory of convective instability (*6*), and the Turing description of reaction-diffusion patterns (*7*) are some examples of this approach, which identifies local and generic structures within the system. However, the systematic derivation of nonlinear field theories remains challenging; not only is reproducing macroscopic behaviors from microscopic patterns difficult, but bridging the gap between macro- and microscopic dynamics is also nontrivial. Instead, abstract symmetries and bifurcation methods are often used to derive the effective field equations with a large number of undetermined parameters (*8*), which limit comparisons with experimental data. Nonetheless, under certain limits of hydrodynamic motion, they remain effective at predicting the formation of local structures that typify a certain configuration of field variables.

In the absence of fluid flow, quasistatic patterns can form on thin films of condensed materials, as can be seen through the buckling and wrinkling of soft elastic membranes (*9*). There are numerous biological examples of these patterns, e.g., the cortical convolutions of the brain (*10*), fingerprint (*11*), and skin wrinkling (*12*). The presence of curvature in biological thin-film wrinkling systems, together with applied stresses, can induce morphogenesis (*13*). Scalar field equations can be derived in cases of quasistatic morphogenesis on soft elastic membranes (*9*) to predict transitions and symmetry-breaking patterns quantitatively. This thus enables targeted engineering of pattern formation on nanosurfaces (*14*) and advances in microlens array fabrication (*15*). In the presence of fluid flow, the governing Navier-Stokes equation and the associated pattern formation dynamics admit complex nonlinearities (movie S1).

Here, we systematically derive and experimentally test an effective asymptotic field theory that quantitatively predicts the surface pattern formation in a curved thin-film fluid system imbued with surface-active materials. Using asymptotic expansion methods under the thin-film lubrication approximation allows for a detailed quantitative analysis of the morphogenesis process as well as predictions of symmetry-breaking and transitional behaviors. A generalized fourth-order equation for the normal displacement field of a curved thin liquid film is derived to the leading order, where the higher-order phenomena, such as the cross-interaction effects between the driving forces and the nonlinear effects of curvature, are assumed to be small. A key motivation for the derivation of this equation is to study the complex and time-dependent process of pattern formation near its excitation for a thin-film system driven by both intrinsic (by expanding local surface curvature terms) (*16*) and surface tension effects (through stress boundary conditions coupled with surface transport) (*17*, *18*). Under conventional full Navier-Stokes models, this is a formidable task with prohibitive computational times even if one overcomes the substantial numerical obstacles involved in such a system characterized by a large range of length and time scales with complex intrinsic properties and boundary conditions. Unphysical numerical artifacts such as parasitic currents (*19*, *20*) and spurious capillary waves (*19*) that arise from difficulties in accurately evaluating curvature numerically can hinder the computational process and are an ongoing topic of current research. The quasistatic asymptotic model considered here allows for an efficient extraction of key insights into the localized pattern-forming instability at a relatively low computational cost. This allows progress to be made in the study of pattern formation instabilities in nonstatic flow systems.

## RESULTS

### Optical flow of bubbles

Because of the transient nature of the morphogenic process (see movie S2), in the long term, the system can be observed (*21*) to tend to either a dot or labyrinth-patterned stable state, similar to the quasistatic case (*9*) or, alternatively, to tend toward a black Newtonian film after undergoing a coarsening process. To examine the effectiveness of the leading-order theory derived, we use appropriate metrics (depending on the degrees of self-similarity exhibited), which quantify the complexity of the patterns of both the experimental images and the simulated result. This provides a baseline result for future studies of similarly transient morphogenic phenomena in a thin liquid film in physiochemical and biophysical systems. For the experimental images, we describe below the method used to obtain the desired phenomenon of pattern formation.

#### *Experiment*

**Fig. 1:**
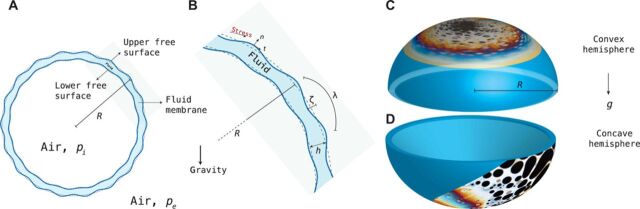
Notation and experimental system. (**A**) Schematic of a curved convex hemispherical thin film of radius *R* and film thickness 2*h*. The air pressure difference between the interior and the exterior of the bubble is given by Δ*p* = *p_e_* − *p_i_*, and *g* is the gravitational acceleration. (**B**) The film is driven toward an instability pattern with wavelength λ and height displacement ζ in the radial direction. (**C** and **D**) Convex and concave hemispherical geometries with the experimental images overlaid at the position where they are observed.

Consider the hemispherical thin-film bubble systems shown in Fig. 1 with a detailed schematic outlined in panels A and B. Using a dilute water-detergent solution, we create stationary hemispherical bubbles in both convex and concave configurations. Letting the bubbles evolve naturally by gravitational drainage, we observe and capture the complex pattern formations, which unfold at the apex regions of the hemispheres. For more details of the experiment, see Materials and Methods, and for details of the optical methods, see section II of the Supplementary Materials.

#### *Thin-film flow velocity*

**Fig. 2:**
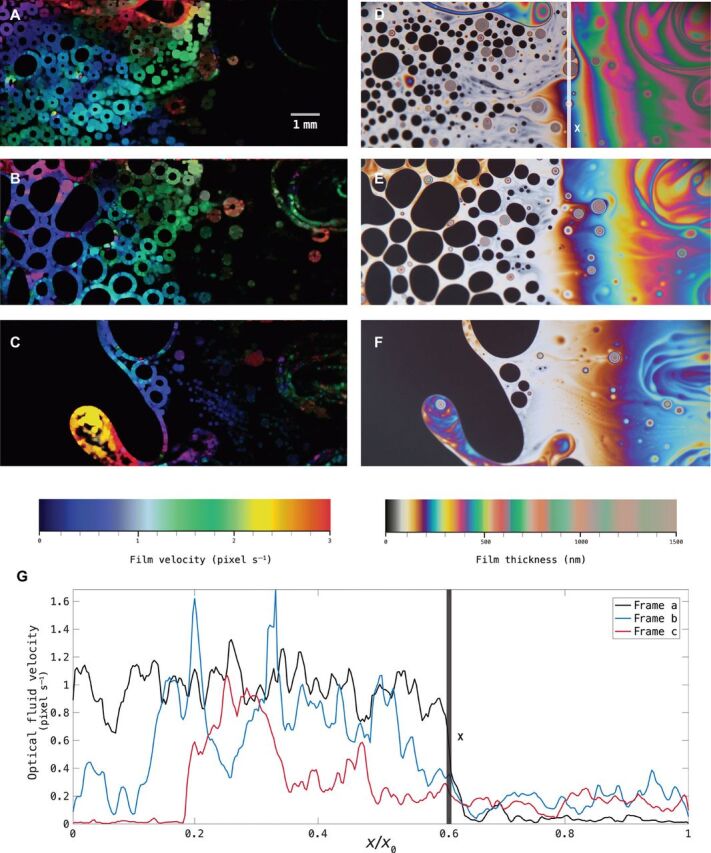
Optimal flow profiles on bubbles. Optical flow profiles (**A** to **C**) of some of the images from the experiments in (**D**) to (**F**). (**G**) shows the evolution of the optical fluid velocity along the horizontal *x* axis (*x*_0_ is the rightmost *x* coordinate of each image), averaged over the vertical axis. Here, 1 pixel corresponds to 1.531 × 10^−4^m in real-life distance. The X line in (D) and (G) marks the transition front between the thin-film flow region on the right and the pattern-forming region on the left.

As a prelude to our general theory of pattern formation on thin films, we first need to confirm that the pattern formation observed differs quantitatively from that of the standard thin-film flow, of which there is vast number of modeling equations in the literature (*22*). Second, we aim to experimentally justify the use of the low Reynolds approximation to drastically simplify our equations of motion. To achieve this, we consider the Reynolds number Re = *uh*/ν with characteristic fluid velocity *u*, the thickness of the film *h* as the characteristic length scale, and kinematic viscosity ν. Since *h* and ν can be measured at the beginning of the dynamics as initial conditions, what remains is to ascertain the order of magnitude of *u*. Experimentally speaking, this is a challenging problem for the thin liquid film, since its transient nature means that it does not tend to remain stable for the amount of time it requires for a complete physical measurement of the flow velocity, nor does the rapid surface transport of surfactants aid with an assessment of the reference as well as the non-equilibrium local surface tension. This calls for the use of an imaging analysis in the form of deep optical flow, which extracts displacement information from adjacent frames (*23*, *24*). Here, we estimate the fluid flow velocities against a known velocity in the slow flowing part of the system shown in Fig. 2 (D to F).

Using an algorithm designed to study the optical flow of motion (*25*), we obtain a matrix of optical flow velocities *I*(*x*, *y*, *t*) for the entire video of the pattern-forming process. Comparing the optical flow velocities *I* with reference real-life velocities captured and computed in the slower-flowing part of the video, we obtain an accurate quantitative measure of the fluid flow velocities across the faster-flowing pattern-forming region. Figure 2 (A to C) shows the optical velocity of the convex thin-film flow experimental configuration. To correlate the optical velocity with the real-life velocity, we examine the slower-flowing (near black) region on the right-hand side of the film. Taking into account the size of the imaging sensor and the magnification ratio, we detect the movement of the colored front in time to calibrate the relation between the optical velocities and the real-life fluid velocities. We found the front marked X on Fig. 2D translated with an upper bound velocity of the order 10^−5^m s^−1^. Furthermore, by tracking the group of small concentric colored ring structures (which denote a localized globule of detergent, the result of surfactants forming agglomerates), we can deduce the surface velocity of the flow to have an upper bound of the order 10^−4^m s^−1^. In comparison, the pattern formation region on the left-hand side of Fig. 2 (D to F) has speed one order of magnitude larger than that of the thin-film flow on the right-hand side (with three orders of magnitude difference observed in extreme cases). However, because of the length scale in this system being on the order of hundreds of nanometers, the low Reynolds approximation Re ≪ 1 can be assumed throughout our experiment.

Moreover, we observe the optical fluid velocity behavior to have two distinct regimes separated by the position X marked on Fig. 2 (D and G). On both sides of the divider, we see divergent behaviors in the (vertically averaged) optical fluid velocity as time progresses. To the right of X, the thin-film regime considered by numerous existing literature (*22*), velocity mostly increases in time, contrary to the pattern-forming region on the left of X where velocity markedly decreases in time. This suggests a substantial break from the conventional thin-film flow and thus requires a different approach to obtaining its equations of motion.

In addition, since the integral of the optical velocity with respect to the horizontal distance is equivalent to the integral of the optical velocity squared (kinetic energy) with respect to time, we note that the areas under the curve in Fig. 2G are numerical representations of the total optical kinetic energy in the system for the corresponding frames and an indication of the evolution of the surface transport velocities as the thin film continues to drain in the direction of the gravity-driven flow. In the next section, we describe the asymptotic method under the low Reynolds approximation, specific to the onset of the instability, to extract the leading-order dynamics from the pattern-forming regime of the overall fluid flow.

### Quasistatic pattern formation on thin films

#### *Asymptotic method*

Starting from the thin-film condition that the film thickness *h* ≪ *R*, where *R* is the bubble radius, the Navier-Stokes equation reduces to the leading order to an amplitude equation through an asymptotic expansion under the limit ϵ → 0, where ϵ = [(M − M_c_)/M*_c_*]^1/2^ is the nondimensional distance to the excitation of the pattern-forming instability, where M = ρ(Δσ)/(μ^2^*k*) and M*_c_* = ρ(Δσ)/(μ^2^*k_c_*) are the Marangoni and critical Marangoni numbers. Here, ρ is the fluid density, Δσ is the gradient in surface tension coefficient, μ is the fluid viscosity, and *k* is the pattern-forming wave number with *k_c_* as its critical value at excitation. This asymptotic method, with more details described below, has been used successfully (*26*) in the analysis of numerous morphogenic processes involving reaction-diffusion species; however, its insight into the pattern formation of nonstatic fluid flow systems is limited, where the coefficients of the amplitude equation are elusive to analytical methods. We found, nevertheless, that a substantial simplification is possible when the flow speed is sufficiently small and the system can be assumed to be quasistatic, as was verified experimentally with the optical flow technique. Hence, we proceed under this crucial simplification to explain analytically such a quasistatic morphogenic system using the asymptotic method derived using the aforementioned approximations.

For a thin-film geometry, the schematics of which is shown in Fig. 1 (A and B), to identify equilibrium configurations and perturb from this equilibrium position to approximate the dynamics of the pattern-forming instability, we assume that the film exhibits an overdamped capillary wave dynamics on the interface. This condition stems from the fact that the instability is observed physically and that the coefficients of the derived amplitude equations are real. However, the converse does not necessarily hold; i.e., being in the overdamped capillary wave regime does not suggest that the instability is triggered. Furthermore, in the presence of surfactant solutions, we can also assume that the interface is sufficiently immobilized (*27*) and that slip effects become negligible on the overall dynamics of the system, and so, we can systematically apply asymptotic expansion to the Navier-Stokes system to obtain, to leading order, the following fourth-order amplitude equation

$$\partial_{\tau }\zeta =\Delta_{2}(c_{1}\zeta +c_{2}\zeta^{2}+c_{3}\zeta^{3}+c_{4}\Delta_{2}\zeta )+c_{5}\zeta +c_{6}\zeta^{2}+c_{7}\zeta^{3}$$ (1)

(details to be found in section I.D of the Supplementary Materials) where Δ_2_ is the two-dimensional Laplace-Beltrami operator, τ = *t*/*t*_0_ is the nondimensional time normalized with the characteristic time scale *t*_0_, and *c*_1_, …, *c*_7_ are coefficients that depend on the Laplace number La = ρσ*h*_0_/μ^2^ and the velocity-independent Marangoni number M = ρ(Δσ)*h*_0_/μ^2^, where *h*_0_ is the initial film thickness. With comparisons to classical pattern-forming systems (*26*), the (*c*_1_, *c*_2_, *c*_3_) terms are responsible for nucleation and the coarsening processes of the instability and the (*c*_5_, *c*_6_, *c*_7_) terms describe dynamics driven by convectional processes, with the *c*_4_ term present in both processes. Henceforth, we shall denote (*c*_1_, *c*_2_, *c*_3_) terms as the coarsening terms and (*c*_5_, *c*_6_, *c*_7_) terms as the convection terms.

The detailed derivation of the asymptotic expansion under the limit ϵ → 0 can be found in sections I.D to I.E of the Supplementary Materials. Assuming linearized boundary conditions around *z* = 0, the orientational and directional independence of ζ, and the quasistatic flow condition, a systematic analysis enables us to express the coefficients in Eq. 1 in terms of the nondimensional parameters La and M, which denote the effect of curvature and the localized strength of the gradient of the surface tension coefficient, respectively. The surface tension near the onset of instability for the initial time is assumed to follow the linear form σ = σ_0_ − *a*Γ, where *a* = −∣∂σ/∂Γ∣ is a gradient of surface tension coefficient with respect to the surfactant concentration Γ and σ_0_ denotes the initial surface tension coefficient. The real coefficients in the amplitude equation (with derivation detailed in section I.G of the Supplementary Materials) are shown in Table 1. These coefficients together with Eq. 1 give a complete leading-order description of the transient pattern-forming dynamics in the overdamped capillary wave regime under quasistatic fluid flow.

**Table 1:** List of normalized parameters for Eq. 1, where $\hat{L}={\text{La}}^{-2}$ and $\hat{M}=M^{-2}$.

$c_{1}=\frac{2}{15}\hat{M}-\hat{L}$
$c_{2}=-\frac{3}{4}\hat{L}$
$c_{3}=\frac{117}{80}-\hat{L}$
$c_{4}=-\frac{38}{75}+\frac{6}{15}\hat{L}$
$c_{5}=\frac{1}{3}\hat{M}$
$c_{6}=\frac{1}{4}\hat{M}$
$c_{7}=\frac{19}{45}\hat{M}+\frac{2}{5}\hat{L}-\frac{28}{75}$

### Pattern excitation frequency

After deriving the amplitude equation of the system, it is now important to determine the point at which the pattern-forming instability is triggered, since the precise determination of the onset of the instability provides insight into the transition points in the dynamics of the system. To achieve this, we decompose the thin-film system into Fourier modes and thus consider this problem using capillary waves and finding the relationship between the instability wavelengths with measurable quantities such as the film fluctuations.

In absence of surfactants, the critical damping wavelength of the system, where the capillary wave transitions from an underdamped to an overdamped regime, is given by $\lambda_{c}^{w}=2\pi l_{\text{vc}}/\epsilon^{⋆2}$, where *l*_vc_ = μ^2^/(ρσ) is the viscocapillary length scale (*28*) and ϵ^⋆^ ≃ 1.3115 (*29*). For soap films under consideration, the presence of the surfactant solution damps the surface waves of the system and thus increases the value of $\lambda_{c}^{w}$ to within the range of the interferometry techniques used to measure the thickness of thin films and the experimentally observed film thickness in the current study.

The dispersion relation of the system in the presence of surfactant solutions was recently (*29*) derived as

$$W_{0}(\omega^{\prime },\varepsilon )(2+\Phi (\omega^{\prime },\varepsilon )+\frac{\beta }{i\varepsilon \omega^{\prime }})+\frac{\beta {\left[\Phi (\omega^{\prime },\varepsilon )\right]}^{1/2}}{i\varepsilon \omega^{\prime }}=0$$ (2)

where *W*_0_(ω′, ε) = 1 + (iω′ + 2ε)^2^ + 4ε^2^(2 + Φ(ω′, ε))^1/2^ is the dispersion relation of the system in the absence of surfactant solutions, for ε = ν*k*^2^/ω_0_, β = *a*Γ_0_*k*/μω_0_, Φ(ω′, ε) = (1 + iω′/ε)^1/2^ − 1, and ω′ = ω/ω_0_, where ω_0_ = (σ*k*^3^/ρ)^1/2^ is the frequency of capillary waves in an ideal fluid.

Since the pattern formation under consideration is observed physically, a necessary condition is that the capillary wave must reside within the overdamped regime after excitation. Hence, we consider that the wavelength of the pattern-forming instability λ is proportional to the critical damping wavelength $\lambda_{c}^{w}$. This provides the relevant length scale estimation for the critical wavelength λ_c_ at the excitation of the pattern-forming instability, with $\lambda ∼\lambda_{c}^{w}$.

To relate the pattern-forming wavelength with the normal surface displacement amplitude, we consider the condition that the fluid velocity be bounded in the region (see section I.G of the Supplementary Materials) as the local film thickness *z* → 0 leads to the power-law relation

$$\overset{–}{\lambda }∼{\overset{–}{\zeta }}^{2/3}$$ (3)

where the nondimensional normal displacement amplitude and the wavelength of the pattern-forming instability are given by $\overset{–}{\zeta }=\zeta /\zeta_{0}$ and $\overset{–}{\lambda }=\lambda /\lambda_{0}$, respectively, where ζ_0_ is the normal displacement amplitude measured at the initial point of the dynamics and λ_0_ is the reference wavelength calculated from the dispersion relation in Eq. 2. Crucially, this relation links the measurable quantity ζ with the theoretical quantity λ, which allows for potentially very accurate predictions of the pattern excitation frequency and wavelength should a robust measurement of the displacement amplitude ζ be made. This paves the way for a complete classification of fluid instabilities in terms of their surface fluctuations, which may be observed experimentally.

#### *Transient pattern stability*

**Fig. 3:**
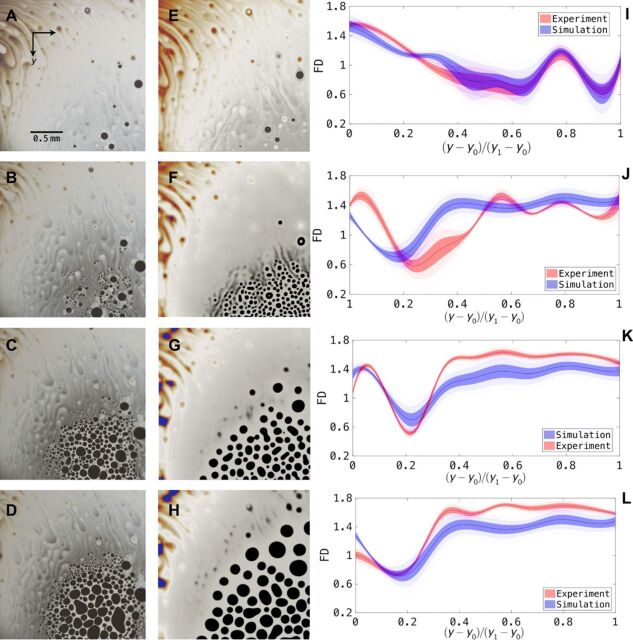
Pattern formation on concave bubbles. Experimental images (**A** to **D**) and simulation results (**E** to **H**) of pattern formation on a concave thin film near rupture. (**I**) to (**L**) show the numerical FDs of (A) to (H), where *y*_0_ and *y*_1_ are the *y* values for the top and bottom edge, respectively. The contours around the curve denote the standard deviations (SD) of the FD, with the deepest color showing the bounds of (1/2) SD and the faded color giving the 1 SD bounds. The relative fractal error δ from top to bottom is as follows: 0.0417, 0.1636, 0.1716, and 0.0926. The colors in (A) to (H) represent film thickness using the same scale as in Fig. 2.

**Fig. 4:**
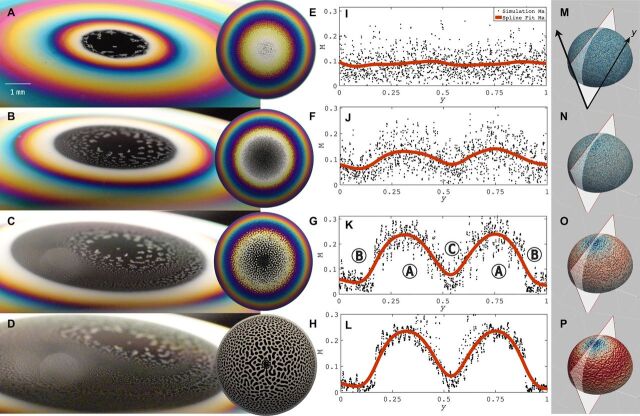
Pattern formation on convex bubbles. Experimental (**A** to **D**) and simulation (**E** to **H**) images of pattern formation on a curved thin film (showing the time-dependent amplitude of the surface fluctuations) with associated (**I** to **L**) plots of the Marangoni number and the Marangoni field (**M** to **P**) on the hemisphere, where *y*_0_ and *y*_1_ are the leftmost and rightmost *y* values of the cross section shown in (M), respectively. In (K), regions A, B, and C refer to the pattern-forming, black-film, and thin-film flow regions, respectively.

Having excited the pattern formation and estimated the triggering point via the critical wavelength of the capillary waves, the stability question of the consequent pattern follows naturally. Since the pattern selection and coarsening at the excitation of the instability are heavily nonlinear processes, the complete stability cannot be derived by a linear analysis. Furthermore, the pattern instability is highly transient and numerical simulations of Eq. 1 in the parameter space of M and La do not yield steady states for all time, but transitions between patterns of different types can be observed as well as patterns of a mixed variety. Figure 3 shows pattern formation on concave bubbles, and Fig. 4 the pattern formation on convex bubbles.

**Fig. 5:**
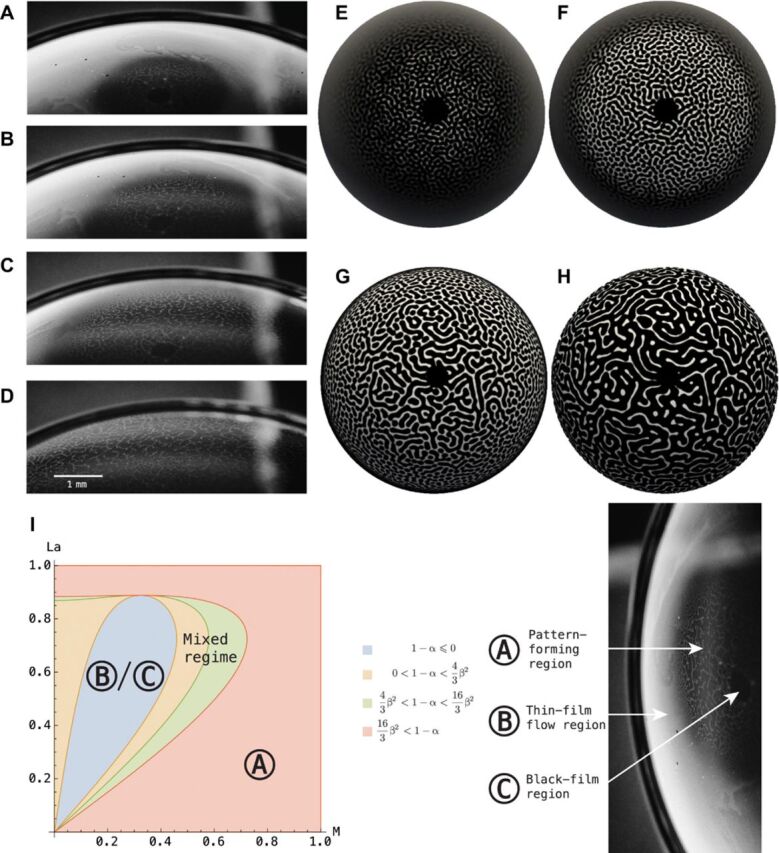
Pattern formation on convex bubbles II and stability diagram. (**A** to **D**) Experimental images of the labyrinth state on a convex hemispherical thin-film system. (**E** to **H**) FEM simulation of the amplitude equation (Eq. 4) in region A. (**I**) Plot of the stability diagram, where A denotes regions of pattern formation, and B and C denote the black-film and the thin-film flow regions, respectively.

These transitions can be understood to be the consequence of terms with even parity in Eq. 1, breaking the invariance of the solution under the reflection map Θ(ζ) = − ζ. The coefficients of the symmetry-breaking terms, under the overdamped thin-film approximation, are shown in Table 1 to depend on La and M, and thus we can expect a role for both curvature and Marangoni effect in the symmetry-breaking transition. In similar symmetry-breaking behavior in the classical convection-driven pattern-forming systems (*9*, *26*, *31*) where the inclusion of similar terms destroys the Θ invariance of the solution, a qualitative comparison suggests that small values of the curvature La and the surface tension gradient M would result in a hexagonal dot phase such as those observed in Fig. 3, while larger values of La and M would yield a labyrinth phase such as those seen in Fig. 5.

The amplitude equation (Eq. 1) can be recast in the normal form

$$\overset{\cdot }{A}=-\Delta \frac{\mathrm{df}(A)}{\mathrm{dA}}+\Delta^{2}A-\alpha A-\beta A^{2}-A^{3}$$ (4)

where · = γ∂_τ_, $A=\zeta \sqrt{\gamma c_{7}}$, α = *c*_5_γ, $\beta =\sqrt{c_{6}\gamma /|c_{7}|}$, and Δ = (2*c*_3_/∣*c*_1_∣)Δ_2_ for $\gamma =4c_{4}/c_{1}^{2}$. In the concave hemisphere experiments, such as the one shown in Fig. 3, the pattern formation exhibits a coarsening behavior, whereas the convex hemisphere, such as the case shown in Fig. 3, has a convection-driven behavior. Under this convection-driven regime, weakly nonlinear stability analysis (*30*) can yield exact regions of the nondimensional parameters $\hat{L}$ and $\hat{M}$ for which different patterns are stable. By comparing the convection-driven terms of the amplitude Eq. 4, i.e., neglecting the coarsening-driven terms, one finds the stability criteria

$$\begin{array}{cccc} \text{Hexagonaldots}: & -\beta^{2}/15 & <1-\alpha < & 4\beta^{2}/3\\ \text{Mixed dots}/\text{labyrinth}: & 4\beta^{2}/3 & <1-\alpha < & 16\beta^{2}/3\\ \text{Labyrinth}: & 16\beta^{2}/3 & <1-\alpha & \end{array}$$ (5)

where $\alpha =\frac{4}{3}\Xi^{2}\hat{M}$, $\beta =\Xi \hat{M}{\left(\frac{13}{15}\hat{M}+\frac{4}{15}\hat{L}+\frac{56}{225}\right)}^{-1/2},$ and Ξ is defined by the relation $\Xi^{2}{\left(\frac{2}{15}\hat{M}-\hat{L}\right)}^{2}=-\frac{38}{75}+\frac{6}{15}\hat{L}$. This criterion is visualized in Fig. 5I.

### Transient structures

For the transient structures we observe in the experimental images in Figs. 4 and 5, understanding its properties allows us to gauge the success of the leading-order asymptotic theory derived. Below, we use geometric metrics to quantify the shape complexity of the evolving structure and also identify an interesting black-hole region at the top of the hemisphere, which can be explained in the framework of the Marangoni field shown in Fig. 4.

#### *Fractal dimensions*

In Fig. 3, we have a localized system of a concave thin-film configuration, whereby there is a gradient of film thickness formed by the gravitational drainage flow (see movie S2). We observe that the theory closely mimics the experimental data qualitatively in terms of the onset and initial evolution of the dot pattern. This suggests that, while the amplitude equation (Eq. 1) is a leading-order approximation of the system, it nonetheless captures the key behavior of the system where the diffusion behaviors dominate over convection in the amplitude equation (see movie S4). We see in Fig. 3 (D and H) that the theory starts to deviate from the experimental results as the higher-order behaviors and convection flows due to localized gradients become relevant.

The challenge is now to quantitatively show the difference in pattern complexities of the simulation and the experimental images shown in Fig. 3 (A to H). A feature observed from the experimental images, which is absent from the simulation, is that of a self-similar property of the patterns as they (the patterns) propagate in space. This feature yields itself to a fractal dimension (FD) analysis to quantify the complexity of the pattern formation. This approach is used in the literature in numerous fields, ranging from clustering large data sets (*32*) and surface roughness problems (*33*) to computer vision problems of texture segmentation (*34*).

To show the finer differences between the experimental images and the simulation results in Fig. 3 (E to L) in a quantitative manner, we numerically obtain the (Minkowski-Bouligand) FD of the image divided into *N* vertical segments, where the FD of a region Ω is defined by ${\text{lim}}_{\delta \to 0}\text{log}\;N_{\delta }/\text{log}\;(1/\delta )$, for *N*_δ_ is the minimum number of sets with diameter of at least δ required to cover Ω. Figure 3 (K and L) shows a consistent difference in FD between the experimental and simulation results later in the pattern-forming evolution. More numerically, we define the relative fractal error δ to satisfy the relation

$$\delta \int_{\Omega }{\text{FD}}^{\text{exp}}=\int_{\Omega }|({\text{FD}}^{\text{exp}}-{\text{FD}}^{\text{sim}})|$$ (6)

over the region Ω, where FD^exp^ and FD^sim^ denote the FDs of the experimental and simulation images, respectively. Integrated over the entire imaging region Ω, this quantifies the relative differences between the full nonlinear system and the leading-order asymptotic model, which captures all but the finest details of the microscale behavior in the pattern formation dynamics. As stated in the caption of Fig. 3, we note that the value of δ is relatively larger in the transient stages of the pattern formation as shown in Fig. 3 (J and K) than the initial and the final stages in Fig. 3 (I and L). This suggests that the first-order asymptotics theory captures the initial evolution as well as the quasisteady stages of the dynamics much better than the intermediate stages. This is consistent with the quasistatic nature of the model, since both the beginning and the end of the dynamics are also relatively static as compared to the intermediate stages.

#### *Pattern formation front*

The propagating front of the pattern formation in Fig. 3 (A to D) is observed to have both a higher density and nonuniformity of dot patterns than regions in its interior. These features, not captured by an equation with coefficients obtained via the leading-order quasistatic approximations as those shown in Table 1, suggest that the front evolves under a different length scale and potentially a different (or even time-dependent) *c_i_* coefficient set in the amplitude equation (Eq. 1). This is not the case for regions away from the front of the pattern formation, which can be due to higher-order fluid flow and surfactant transport terms neglected in the leading-order approximation, consequently causing subtle variations in film thickness and properties in the propagating front region, thereby resulting in a different length scale and pattern features. Another observation is that in Fig. 3B, the multiscale or fractal nature of the nucleation process in the experiment is not well captured in the leading-order model, although the nucleation sites are well identified. This is partly reflected in the discrepancies in the FD plots in Fig. 3J. A more sophisticated method is required to deal with these finer differences, which is beyond the scope of a leading-order method presented in this contribution.

#### *Pattern transitions and the Marangoni field*

A particular case in which the coarsening and convection-driven features manifest simultaneously is shown in Fig. 4 in a convex hemispherical thin-film system. For this system, we observe multiple instability fronts where the pattern formation switches from a configuration of uniform hexagonal dots to one of labyrinth state (see movies S3 and S4). We deduce from this that the local pattern formation is a function of not only the film thickness initial condition but also the local values of the Marangoni (M) and Laplace (La) numbers. Assuming that the local curvature does not deviate from the initial condition in the particular case as shown in Fig. 4 (A to D), then the Marangoni number is an indicator of the state of the pattern formation. We consider here the Marangoni number calculated from the simulation results in Fig. 4 (I to L). Moreover, a slice of the Marangoni number across the hemisphere from the simulation in Fig. 4 (I to L) shows that the peak values of the M correspond to regions of labyrinth behavior, whereas trough values denote hexagonal states. Physically, this suggests that a greater local variation of the surface tension necessarily leads to a more disordered and symmetry-breaking pattern formation, whereas a smaller local perturbation in the local surface tension creates the more orderly and symmetrical hexagonal state of pattern formation.

#### *Black-hole region*

Another curious feature that is observed in a convex hemispherical thin-film system, as shown in Fig. 5 (A to D) as well as movies S3 and S4, is the appearance of a circular “black hole” region at the topmost part of the hemisphere, corresponding to the thinnest portion of the liquid film. The same phenomenon is also observed in Fig. 4 (A and B).

We denote this the black-film region C in Fig. 5I, wherein the molecules at the interface are in a close packing configuration (*35*). Locally, this close packing correlates to a fully adsorbed interface, which results in the homogeneous condition that there is negligible Marangoni gradient, i.e., M ≪ 1. In terms of the amplitude equation, the black-film region C admits a U-shaped energy potential *f*(A) in Eq. 1, which can be linearized to the Helmholtz form (Δ_2_ + Ω^2^)ζ = 0, where the film thickness ζ can be interpreted in this case as the amplitude of time-harmonic solutions to the wave equation η*_tt_* = Δη, with frequency Ω given by Ω^2^ = *c*_5_/*c*_1_ for η = ζexp ( − *i*Ω*t*). The boundary condition is given by the thickness-wavelength relation in Eq. 3 that ${\text{lim}}_{\zeta \to \zeta_{c}}\eta (r_{c},\theta ,t)=0$, where $\zeta_{c}\sim {\left(\lambda_{c}^{w}\right)}^{3/2}$ and *r*_c_ is the radius from the apex where the film has thickness ζ_c_. This is similar to the classical vibrating drumskin problem (*40*) whose circumference is held in a plane. This is the limiting case for a fluidic membrane where the fluid has been drained and the film becomes a surfactant bilayer. The results shown in Fig. 5 (E to H) depict the evolution of the black-hole region upon imposition of a Gaussian decay condition on the full amplitude equation at the topmost region of the spherical cap characterized by a radius *r* = *r*_cap_ ≪ 1. This is to avoid the presence of a numerical singularity.

As $\zeta \to \zeta_{c}^{+}$, M = *O*(1), the patterns correspond to the peaks of the Marangoni number plot, also referred to as the pattern-forming region A in Figs. 4K and 5I, and evolve according to the amplitude equation (Eq. 1) with a W-shaped quartic energy potential *f*(A). Last, in the thin-film region B, the dominant dynamics is the thin-film fluid flow rather than the pattern-forming instability, and this is shown as M reaches a trough in Fig. 4K. In contrast to the black-film region C, the local Marangoni gradient is negligible due to a lack of coverage of surfactant on the interface as the fluid flow transports surface materials along the interface. Correspondingly, in Fig. 4 (M to P), we observe that this interfacial transport process congregates the surfactants near the top of the hemisphere as time increases, reinforcing the idea of a two-regime dynamics, where the surfactant-concentrated region exhibits quasielastic (*9*) pattern formation behaviors, while thin-film fluid flow is the dominant behavior for regions where surfactant concentration is low.

## DISCUSSION

We demonstrated that an effective field theory derived systematically using asymptotics provides a good quasiquantitative description of the surface pattern formation in nonplanar viscous thin liquid films in the presence of a surfactant solution. Systems that exhibit similar pattern-forming capabilities can be found across different length scales, but the underlying principle of symmetry breaking in the field equation is a common mechanism by which patterns form on the surface of a homogeneous film, and the presence of surfactants along with geometric curvature provides inputs and enables a more systematic classification of the overall wrinkling process in a dynamic situation. Thus, the proposed analytical approach can help us to recognize pattern formation processes more quantitatively beyond a static situation and is an important step toward completely characterizing the complex dynamic behavior of the thin film near the process of rupture to the leading order. A by-product of the verification of the Stokes regime of the equation of motion via the technique of dense optical flow using video imagery gives us a previously unidentified method to determine film flow speeds at length scales, which are traditionally inaccessible to the placement of sensors and instruments.

Another interesting result of this analytical formulation is that the thin film naturally yields a power relation between film thickness and the instability wavelength λ through the requirement that local fluid velocities are finite. In addition, the proximity of the critical instability wavelength λ*_c_* to the critical damping wavelength of capillary waves, $\lambda_{c}^{w}$, as observed experimentally with interferometry, together with previous literature on capillary waves in the presence of surfactants (*29*, *30*), prompts a relation between the two quantities. We expect the capillary wave theory together with a more sensitive treatment of transient film compositions to be instrumental in understanding film behavior and the nucleation of black spots (*36*) near the rupture process and that the capillary wave theory provides a useful vehicle through which we can understand the damping behaviors and the roughness of the interface in the neighborhood of the onset of the pattern-forming instability. We are encouraged by recent results in pattern formation for stationary elastic spherical objects (*9*) and we anticipate that our work will lead to further experimental studies in the measurement of the relevant quantities in transient and fluidic pattern-forming phenomena, such as the amplitude of the capillary waves, the fractal behaviors near the propagating front of pattern formation, and the precise distributions of surfactant solutions. These advances, in turn, will contribute to the solution of long-standing issues in thin films and bubble dynamics and their many applications.

## MATERIALS AND METHODS

### Experimental method

A dilute water-detergent solution with 3% volume detergent (Fairy liquid, Procter and Gamble) is used to create the bubble under room temperature. In the convex hemispherical case, a large bubble (of radius more than 5 cm) is created on the surface of a bath of the dilute water-detergent solution and the camera is trained on the uppermost portion of the bubble as illustrated in Fig. 1C. The full setup and optics are shown in section II of the Supplementary Materials. For the concave case, a hemispherical bubble is stabilized between a fixed circular plastic ring of 2.5 cm in radius, and the camera is focused on the bottommost portion of the bubble, as shown in Fig. 1D, where the pattern formation is superimposed in an exaggerated manner on the hemispherical bubble to show where the camera is pointing. A digital single-lens reflex camera with an APS-C CMOS (advanced photo system type-C complementary metal-oxide semiconductor) sensor (Nikon D500) and a 1:1 macrolens [Nikkor 105mm f/2.8 VR (vibration reduction) AF-S (auto focus with silent wave motor) macro] is placed in front of a bath of soap solution, which is lit in front with a light source. The resulting footage (captured at 24 frames per second at 4K, i.e., 8 megapixel resolution) on the apex of the soap bubble is then edited in the Adobe Lightroom software for white balance.

Assuming that the hemispherical bubbles have a radius of 5 and 2.5 cm in the convex and concave cases, respectively, the pattern-forming regions captured here account for less than a 10th of the total area of the hemisphere. Furthermore, since the pattern formation occurs at the apex of the hemispheres, we shall neglect any edge and contact line effects. After the bubble is formed (on the water-detergent bath in the convex case and on the plastic ring in the concave case), it is allowed to evolve undisturbed and the resulting dynamics are captured.

### Algorithm

The simulation of the amplitude equation in Eq. 1 is nontrivial. While the double-well potential is reminiscent of the Cahn-Hilliard equation, the polynomial terms are a feature of the Swift-Hohenberg equation, coupled with the fourth-order bi-Laplacian operator. This presents challenges within the finite element method. Using a Galerkin method requires either a piecewise smooth and globally *C*^1^-continuous element for a basis function or a mixed formulation that bypasses the *C*^1^-continuous requirement by introducing an auxiliary field to recast the fourth-order equation of motion into two coupled second-order equations. Here, we used the mixed formulation that has been shown, for Cahn-Hilliard class equations (*37*), to be less computationally expensive with a comparable accuracy to *C*^1^-continuous methods. To realize the finite element formulation, the solution of a system state is given via the interpolation function $A(x)=\sum_{i=1}^{N}A_{i}N_{i}(x),$ where *N_i_* are the finite-element basis and *A_i_* are the coefficient for each triangular elements with index *i* = 1, …, *N*. The mesh used for the Galerkin projection is a triangulation of the interface with 97,000 elements for the (convex) hemispherical case and 87,000 elements for the (quasi-)planar concave case. The FEM (finite element method) package FENiCs (*38*) is used for the calculation, whereby integrals over the manifold mesh are reduced to integrals over the surface facets of a mesh (*39*). The Crank-Nicolson method is used for the discretization in time, and the Newton-Krylov solvers based on PETSc’s (portable, extensible toolkit for scientific computation) SNES (scalable nonlinear equations solvers) module are used with the discretizations in space and time solved using the general minimal residual method. Each iteration is solved to a relative tolerance of 10^−6^. The solution process scales well with multiple cores using the MPI (message passing interface) routine.

## Supplementary Material

abb0597_SM.pdf

abb0597_Movie_S4.mp4

abb0597_Movie_S3.mp4

abb0597_Movie_S2.mp4

abb0597_Movie_S1.mp4

## SUPPLEMENTARY MATERIALS

Supplementary material for this article is available at http://advances.sciencemag.org/cgi/content/full/6/28/eabb0597/DC1
