# Supplementary material for: Transient structures in rupturing thin films: Marangoni-induced symmetry-breaking pattern formation in viscous fluids
Source: Sci Adv. 2020 Jul 8;6(28):eabb0597. doi: 10.1126/sciadv.abb0597 (PMC7343401; doi:10.1126/sciadv.abb0597)
Supplement: abb0597_SM.pdf [file abb0597_SM.pdf]

Science Advances
AAAS

advances.sciencemag.org/cgi/content/full/6/28/eabb0597/DC1

Supplementary Materials for

Transient structures in rupturing thin films: Marangoni-induced symmetry-breaking pattern formation in viscous fluids

Li Shen*, Fabian Denner, Neal Morgan, Berend van Wachem, Daniele Dini

*Corresponding author. Email: l.shen14@imperial.ac.uk

Published 8 July 2020, *Sci. Adv.* **6**, eabb0597 (2020)
DOI: 10.1126/sciadv.abb0597

The PDF file includes:

Supplementary Text
Figs. S1 to S3
References

Other Supplementary Material for this manuscript includes the following:

(available at advances.sciencemag.org/cgi/content/full/6/28/eabb0597/DC1)

Movies S1 to S4

In this Supplementary Material, we derive an effective theory for the pattern formation of curved thin liquid films in the presence of a surfactant solution. Starting from the Navier-Stokes equation, we show using asymptotic expansion that the fluid dynamics can be reduced to a generalised hybrid Swift-Hohenberg-Cahn-Hilliard equation. The coefficients of this equation are obtained with an overdamped assumption in the lubrication regime, under which a power-law relation is derived between the film thickness and the wavelength of the instability. Using weakly-nonlinear analysis of this hybrid fourth-order partial differential equation, we predict transient morphogenesis for labyrinth and (hexagonal) dot patterns depending on the local curvature and Marangoni number.

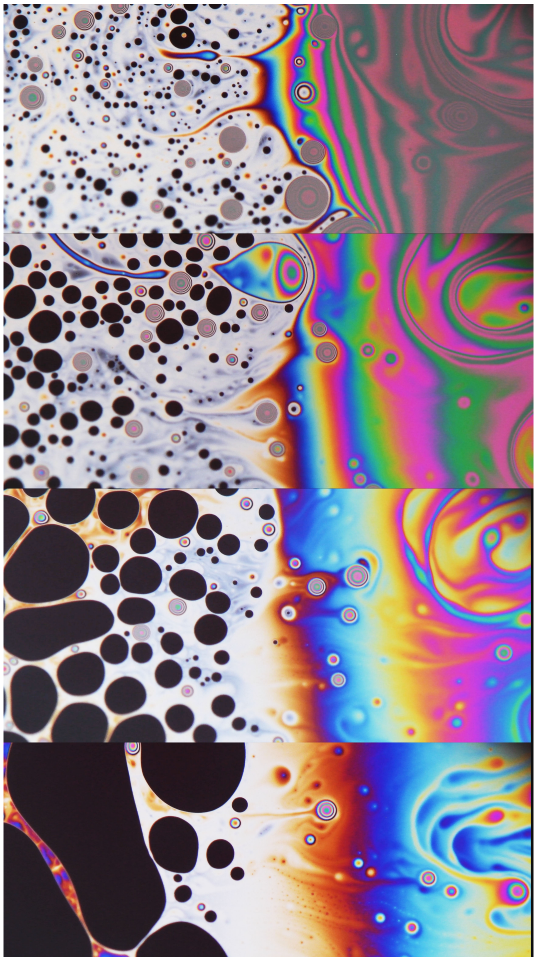

S1. **Pattern formations on a bubble.** Experimentally observed pattern formation and evolution (top to bottom) on a soap bubble film.

MORPHOGENESIS ON A BUBBLE

Turing patterns [7], the natural formation of strips and spots patterns from a homogeneous and uniform state occur in many biological systems [9, 31]. In this Supplementary Material, we examine the curious case of the Turing-like pattern formation on curved thin films in the presence of surfactants as shown in figure (1) and detail the derivation of the leading-order amplitude equation which governs much of the dynamics of the pattern formation using asymptotic theory.

I. ASYMPTOTIC EXPANSION

The (Navier-)Stokes equation describes the motion of the fluid in the viscous membrane. We extend this theory in the thin-region using asymptotics to yield an amplitude equation to describe the resulting pattern formation instability that occur below a certain critical wavelength.

We model the soap bubble as a thin film surrounded by two free liquid interfaces as shown in figure (2a) with pressure difference $\Delta p = p_i - p_e$, normal and tangent vectors $\mathbf{n}$ and $\mathbf{t}$. In the zoomed-in figure (2b), we note that the interface admits a wave-form of height ζ and wavelength λ on application of stress in the tangential direction due to external forces such as the Marangoni effect or curvature. In this Supplementary Material, we shall use ζ for interface height in the pattern formation sections and h for the film thickness in the thin-film flow sections. In the next subsection, we consider the equations of motion in the thin-film system.

A. Equations of motion

Consider an infinite, thin horizontal layer of viscous, incompressible fluid subject to gravity g and a surface-active solution of concentration Γ. The lower boundary lies on a rigid plane while the upper boundary is a free surface $z = \zeta(x, y, t)$. The setup is then reflected to yield the dual-interface thin-film configuration as shown in figure (2). For regions with low Reynolds number, the fluid velocity $\mathbf{u} = \mathbf{u}_\perp + w\mathbf{e}_z$, where $\cdot_\perp$ represents planar quantities with $\mathbf{u}_\perp = (u, v)$, satisfies the time-dependent Stokes' equation

$$\partial_t \mathbf{u} + (\mathbf{u} \cdot \nabla)\, \mathbf{u} = -\frac{1}{\rho} \nabla p + \nu \Delta \mathbf{u} \tag{1}$$

$$\nabla \cdot \mathbf{u} = 0 \tag{2}$$

with streamwise boundary condition $\mathbf{u} = \mathbf{U}$ at the free surface and the interfacial conditions

$$\frac{\mathrm{D}\zeta}{\mathrm{D}t} \equiv \left(\frac{\partial}{\partial t} + u \frac{\partial}{\partial x} + v \frac{\partial}{\partial y} \right) \zeta = w \tag{3}$$

$$\mathbf{t}_i \cdot \mathbf{T} \cdot \mathbf{n}|_-^+ = \mathbf{t}_i \cdot \nabla \sigma \tag{4}$$

$$\mathbf{n} \cdot \mathbf{T} \cdot \mathbf{n}|_-^+ = \sigma \kappa \tag{5}$$

at $z = \zeta(x, y, t)$, where we define the linearised normal $\mathbf{n} = (-\zeta_x, -\zeta_y, 1)/\|\mathbf{n}\|$ and tangents $\mathbf{t}_1 = (1, 0, \zeta_x)/\|\mathbf{t}_1\|$,

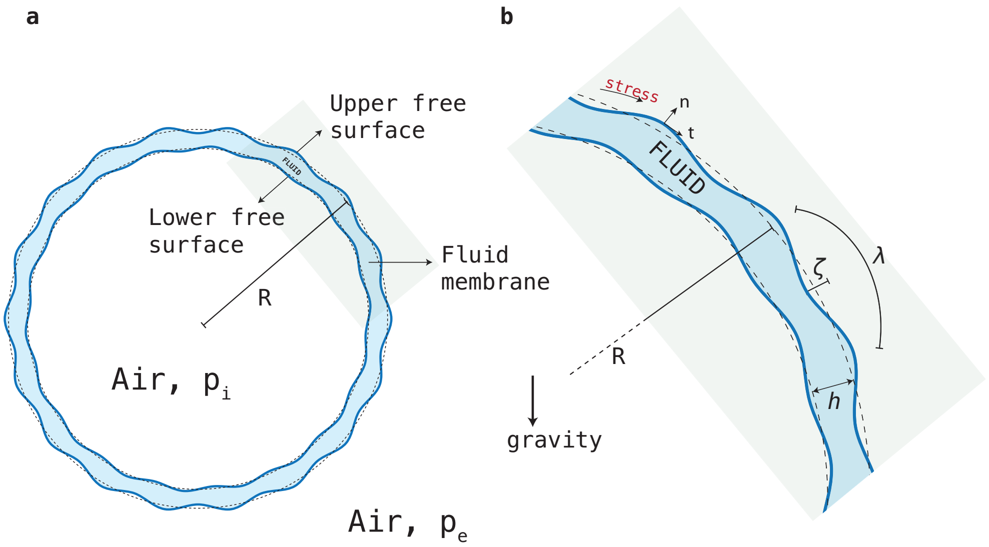

S2. **Schematic of bubble interface.** a. Dual-interface configuration of soap bubble of radius R, interface height ζ, wavelength λ, normal and tangent vectors $\mathbf{n}, \mathbf{t}$ and internal and external pressures p_i and p_e, respectively. b. zoomed-in view of interface.

$\mathbf{t}_2 = (0, 1, \zeta_y)/\|\mathbf{t}_2\|$, in the x and y direction, respectively. The fluid stress tensor $(\mathbf{T})_{ij} \equiv T_{ij}$ is given by

$$T_{ij} = -p\delta_{ij} + 2\mu e_{ij} \tag{6}$$

where δ_{ij} is the Kronecker delta and $(\mathbf{E})_{ij} \equiv e_{ij}$ is the strain rate tensor defined by

$$e_{ij} = \tfrac{1}{2}(\partial_j u_i + \partial_i u_j), \tag{7}$$

written

$$\mathbf{E} = \frac{1}{2}\begin{pmatrix} 2u_x & u_y + v_x & w_x + u_z \\ u_y + v_x & 2v_y & w_y + v_z \\ w_x + u_z & w_y + v_z & 2w_z \end{pmatrix}, \tag{8}$$

where we have used the notation $\partial_j u_i = \partial u_i/\partial x_j$. Eliminating pressure from (1) by taking $-\nabla\times\nabla\times$ and using the identity $\nabla\times\nabla\times\mathbf{u} = \nabla(\nabla\cdot\mathbf{u}) - \Delta\mathbf{u}$ and (2) gives

$$\partial_t \Delta\mathbf{u} - \nu\Delta^2\mathbf{u} = \nabla\times\nabla\times(\mathbf{u}\cdot\nabla\mathbf{u}) \tag{9}$$

The velocity components must be continuous across the interface, so at $z = h$, we have $[\mathbf{u}] = 0$, where $[\cdot]$ denotes the jump of the value of $\cdot$ across the interface.

The surfactant concentration Γ along a deforming 2-dimensional interface satisfies the transport equation [41]

$$\frac{\partial\Gamma}{\partial t} + \mathbf{u}_s\cdot\boldsymbol{\nabla}_s\Gamma - \kappa\Gamma(\mathbf{u}\cdot\mathbf{n}) = D_s\Delta_s\Gamma, \tag{10}$$

where D_s is the surfactant diffusivity coefficient and κ is the mean curvature of the interface. Relating Γ to the surface tension σ, we consider the linear equation of state

$$\sigma = \sigma_0 - \alpha(\Gamma - \Gamma_0) \tag{11}$$

where $\alpha = \mathrm{d}\sigma/\mathrm{d}\Gamma$, and σ_0, Γ_0 are the initial surface tension and surfactant concentration, respectively. Hence $\Delta\sigma = \alpha(\Gamma_0 - \Gamma_\infty)$ is the surface tension difference between the initial position and the infinity position far away from the interface. We are not limited to a singular source of Marangoni gradients. Indeed, the formulation allows a generalisation to different sources such as thermal and compositional effects.

Additional remarks. Since the air-water interface is sufficiently immobilised by surfactants [27], we can assume slip effects to have negligible influence on the dynamics of the interface. In cases of a fully mobile interface in absence of surfactants, slip effects should be considered.

B. Dimensionless number of relevance

Let L, U, T be reference values for length, fluid velocity and timescale, define the non-dimensional velocity

$$\mathbf{u}^*(\mathbf{x}^*, t^*, \sigma^*, p^*) \equiv \mathbf{u}\left(\mathbf{x}/L, t/T, \sigma/\Sigma, p^*\right)/U$$

with the viscous pressure scaling $p^* = pL/(\mu U)$. The non-dimensional time-dependent Navier-Stokes' equation reads

$$\frac{\partial\mathbf{u}^*}{\partial t^*} + \mathbf{u}^*\cdot\nabla^*\mathbf{u}^* = -\nabla^* p^* + \Delta^*\mathbf{u}^* \tag{12}$$

$$\nabla^*\cdot\mathbf{u}^* = 0 \tag{13}$$

$$\frac{\partial\Gamma^*}{\partial t^*} + \mathbf{u}_s^*\cdot\nabla_s^*\Gamma^* - \kappa^\star\Gamma^*(\mathbf{u}^*\cdot\mathbf{n}) = \frac{1}{\mathrm{Sc}}\Delta_s^*\Gamma^* \tag{14}$$

with the kinematic and interfacial conditions

$$\left(\frac{\partial}{\partial t^*} + \mathbf{u}^*\cdot\nabla^*\right)\zeta = w^* \tag{15}$$

$$[\mathbf{t}_i\cdot\mathbf{T}^*\cdot\mathbf{n}] = \mathrm{M}\,\mathbf{t}_i\cdot\nabla^*\sigma^* \tag{16}$$

$$[\mathbf{n}\cdot\mathbf{T}^*\cdot\mathbf{n}] = \mathrm{La}\,\kappa - \mathrm{Ga}\,z^* \tag{17}$$

at $z^* = \zeta(x^*, y^*, t^*)$, where $\Gamma^* = \Gamma/\Gamma_0$ is the non-dimensional surfactant distribution, $\kappa^\star = \kappa/L$ and

$$\mathrm{Sc} = \frac{\nu}{D_s}, \tag{18}$$

$$\mathrm{La} = \frac{\rho\sigma L}{\mu^2}, \tag{19}$$

$$\mathrm{Ga} = \frac{\mathrm{g}L^3}{\nu^2}, \tag{20}$$

$$\mathrm{M} = \frac{\rho(\Delta\sigma)L}{\mu^2} \tag{21}$$

are the Schmidt, Laplace, Galilei and Marangoni numbers. We note here that the number $\mathrm{M} \equiv \mathrm{Re}\,\mathrm{Ma}$ is a velocity-independent Marangoni number where the standard Marangoni number is given by $\mathrm{Ma} = \Delta\sigma/(\mu U)$ and the Reynolds number is given by $\mathrm{Re} = \rho UL/\mu$.

Consider the leading-order term of the surface stress

$$\mathbf{T}\cdot\mathbf{n} \sim (\mu(w_x + u_z),\, \mu(w_y + v_z),\, -p + 2\mu w_z). \tag{22}$$

Hence, to the first-order, to eliminate u and v, consider

$$\frac{\partial}{\partial x}(\mathbf{t}_1\cdot\mathbf{T}\cdot\mathbf{n}) + \frac{\partial}{\partial y}(\mathbf{t}_2\cdot\mathbf{T}\cdot\mathbf{n}) = \mathrm{M}\nabla_2\cdot\nabla_2\sigma \tag{23}$$

and the incompressible condition reduces to

$$\left(\Delta_2 - \partial_z^2\right) w + \mathrm{M}\Delta_2\Gamma = 0. \tag{24}$$

C. Scaling

The equations in this work model the dynamics of system at the onset of pattern-forming instability, in this subsection, we define the relevant parameters to be used in the asymptotic expansion.

Firstly, using the velocity-independent Marangoni number M, we define its critical value the *critical Marangoni number* to be

$$\mathrm{M}_c = \frac{\rho(\Delta\sigma)}{\mu^2 k_c} \tag{25}$$

where k_c is the critical wavenumber. This definition allows for the existence the expansion parameter of the system ϵ to satisfy

$$\epsilon^2 = \frac{\mathrm{M} - \mathrm{M}_c}{\mathrm{M}_c}. \tag{26}$$

We interpret ϵ as the non-dimensional distance from the critical Marangoni number at particular point in space and time. When $\epsilon = 0$, the local Marangoni number reaches its critical value and represents the onset of the pattern-forming instability.

Notation note. ϵ is defined in terms of ϵ^2 to tidy up the asymptotic expansion and minimise the appearence of fractional-powered terms.

Secondly, rewriting spatial (isotropic) and temporal variables in terms of the expansion parameter, we have the multiscale expression

$$\begin{pmatrix}(x,y)\\ t\end{pmatrix} = \begin{pmatrix}(X,Y)\\ T\end{pmatrix} + \begin{pmatrix}X'/\epsilon\\ T'/\epsilon^2 + T''/\epsilon^4\end{pmatrix}, \tag{27}$$

where the undashed quantities are fast excitation and the dashed are slower evolutionary timescales in terms of the expansion parameter ϵ. Naturally, to study the behaviour near the onset of the pattern-forming instability, we perturb the system under the limit $\epsilon \to 0$.

Thirdly, we define the system variables $\mathbf{u}, \bar{p}, \zeta, \Gamma$ on which the asymptotic expansion is carried out, namely,

$$(\mathbf{u}, \bar{p}, \zeta, \Gamma) = \epsilon\boldsymbol{\Theta}^{(1)} + \epsilon^2\boldsymbol{\Theta}^{(2)} + \epsilon^3\boldsymbol{\Theta}^{(3)} + \cdots \tag{28}$$

where

$$\boldsymbol{\Theta}^{(i)} = (\mathbf{u}^{(i)}, p^{(i)}, \zeta^{(i)}, \Gamma^{(i)}) \tag{29}$$

is the system vector and $\bar{p} = p - p_0$ is the reduced pressure.

Finally, for use in the later section of the asymptotic expansion, we consider the derivative scalings for the temporal and surface derivatives

$$\begin{pmatrix}\partial_t\\ (\partial_x, \partial_y)\end{pmatrix} = \begin{pmatrix}\partial_T + \epsilon^2\partial_{T'} + \epsilon^4\partial_{T''}\\ (\partial_X, \partial_X) + \epsilon(\partial_{X'}, \partial_{Y'})\end{pmatrix}, \tag{30}$$

and the differential operators, which transforms to

$$\nabla \to (\nabla_\perp, \partial_z) \tag{31}$$

$$\Delta \to (\nabla_\perp, \partial_z)\cdot(\nabla_\perp, \partial_z) = \Delta_\perp + \partial_z^2 \tag{32}$$

$$\Delta^2 \to (\Delta_\perp + \partial_z^2)(\Delta_\perp + \partial_z^2) = \Delta_\perp^2 + 2\Delta_\perp\partial_z^2 + \partial_z^4, \tag{33}$$

where $\nabla_2 = (\partial_X, \partial_Y)$. Under the asymptotic expansion, these differential operators are given by

$$\nabla_\perp \to \nabla_2 + \epsilon\nabla_2', \tag{34}$$

$$\Delta_\perp \to \Delta_2 + 2\epsilon\nabla_2\cdot\nabla_2' + \epsilon\Delta_2', \tag{35}$$

$$\begin{aligned}\Delta_\perp^2 \to &\Delta_2^2 + 4\epsilon\Delta_2\nabla_2\cdot\nabla_2' + 6\epsilon^2\Delta_2\Delta_2' \\ &+ 4\epsilon^3\nabla_2\cdot\nabla_2'\Delta_2' + \epsilon^4(\Delta_2')^2.\end{aligned} \tag{36}$$

D. Asymptotic expansion

Expanding the system of equations in terms of the asymptotic parameter ϵ under the limit $\epsilon \to 0$, we work under the following assumptions:

- Interfacial boundary conditions are linearised to $z = 0$, the curvature term $-\nabla\cdot\mathbf{n}$ is linearised to $\Delta_2\zeta$, as the higher-order terms will not contribute to the overall amplitude equation under consideration in the asymptotic expansion.

- Pattern amplitude ζ is independent of orientation of the interface nor its direction in the plane and so terms in odd powers of $\nabla_2 \cdot \nabla_2'$ are neglected

The Stokes' equation $\partial_t \Delta \mathbf{u} - \Delta^2 \mathbf{u} = 0$ becomes

$$\left[\left(\partial_T + \epsilon^2 \partial_{T'} + \epsilon^4 \partial_{T''}\right)\left(\Delta_2 + \epsilon^2 \Delta_2' + \partial_z^2\right) - \left(\Delta_2^2 + 6\epsilon^2 \Delta_2 \Delta_2' + \epsilon^4 (\Delta_2')^2 + \partial_z^4\right)\right] w = -G_1. \quad (37)$$

The kinematic condition on $z = 0$ is

$$\left(\partial_T + \epsilon^2 \partial_{T'} + \epsilon^4 \partial_{T''}\right)\zeta + \mathbf{u}_\perp \cdot (\nabla_2 + \epsilon \nabla_s')\zeta - w_z = -G_2. \quad (38)$$

The tangential stress condition is

$$(\Delta_2 + \epsilon^2 \Delta_2' - \partial_z^2) w + \mathrm{M}\left(\Delta_2 + \epsilon^2 \Delta_2'\right)\Gamma = -G_3 \quad (39)$$

and the normal condition is

$$\left[3(\Delta_2 + \epsilon^2 \Delta_2') - (\partial_T + \epsilon^2 \partial_{T'} + \epsilon^4 \partial_{T''}) + \partial_z^2\right] w_z + \left[\mathrm{Ga} - \mathrm{La}\left(\Delta_2 + 2\epsilon \nabla_2 \cdot \nabla_2' + \epsilon^2 \Delta_2'\right)\right] \times (\Delta_2 + 2\epsilon \nabla_2 \cdot \nabla_2' + \epsilon^2 \Delta_2')\zeta = -G_4. \quad (40)$$

Finally, the surfactant transport is given by

$$\left(\partial_T + \epsilon^2 \partial_{T'} + \epsilon^4 \partial_{T''} + \mathbf{u}_\perp \cdot (\nabla_2 + \epsilon \nabla_s')\right)\Gamma - \mathrm{Sc}^{-1}\left(\Delta_2 + \epsilon^2 \Delta_2'\right)\Gamma = -G_5. \quad (41)$$

Hence to order $O(\epsilon^m)$, we have the system

$$\left[\partial_T(\Delta_2 + \partial_z^2) - (\Delta_2^2 + \partial_z^4)\right] w^{(m)} = -G_1^{(m)} \quad (42)$$

$$\partial_T h^{(m)} - w^{(m)} = -G_2^{(m)} \quad (43)$$

$$\left(\Delta_2 - \partial_z^2\right) w^{(m)} + \mathrm{M}\,\Delta_2 \Gamma = -G_3^{(m)} \quad (44)$$

$$\left(3\Delta_2 + \partial_z^2 - \partial_T\right) w_z^{(m)} + (\mathrm{Ga} - \mathrm{La}\Delta_2)\,\Delta_2 \zeta^{(m)} = -G_4^{(m)} \quad (45)$$

$$\partial_T \Gamma - \mathrm{Sc}^{-1} \Delta_2 \Gamma^{(m)} = -G_5^{(m)} \quad (46)$$

$$w^{(m)} = w_z^{(m)} = 0 \quad (47)$$

where equations (43), (44) and (45) are defined for $z = 0$ and $G^{(m)}$ are the terms in addition to the linear problem which appears in the system. The non-trivial terms start with order $O(\epsilon^3)$.

For $O(\epsilon^3)$, the relevant additional terms are given by

$$G_1^{(3)} = \left(\partial_T \Delta_2' + \partial_{T'} \Delta - 6\Delta_2 \Delta_2' - \mathrm{C}_{11}\right) w^{(1)} \quad (48)$$

$$G_2^{(3)} = \partial_{T'} \zeta^{(1)} \quad (49)$$

$$G_3^{(3)} = \Delta_2' w^{(1)} + \mathrm{M}\,\Delta_2' \Gamma^{(1)} \quad (50)$$

$$G_4^{(3)} = \left(3\Delta_2' - \partial_{T'}\right) w_z^{(1)} + \left(\mathrm{Ga} - 6\mathrm{La}\Delta_2\right) \Delta_2' \zeta^{(1)} \quad (51)$$

$$G_5^{(3)} = \partial_{T'} \Gamma^{(1)} - \mathrm{Sc}^{-1} \Delta_2' \Gamma^{(1)}. \quad (52)$$

For $O(\epsilon^5)$, we have

$$G_1^{(5)} = \left(\partial_{T''} \Delta + \partial_{T'} \Delta_2' - \Delta_2^{2'} - \mathrm{C}_{31}\right) w^{(1)} + \left(\partial_T \Delta_2' + \partial_{T'} \Delta - 6\Delta_2 \Delta_2' - \mathrm{C}_{13}\right) w^{(3)} \quad (53)$$

$$G_2^{(5)} = -\partial_{T''} \zeta^{(1)} + \partial_{T'} \zeta^{(3)} \quad (54)$$

$$G_3^{(5)} = \Delta_2' w^{(3)} + \mathrm{M}\,\Delta_2' \Gamma^{(3)} \quad (55)$$

$$G_4^{(5)} = -\partial_{T''} w_z^{(1)} - \mathrm{La}\Delta_2^{2'} \zeta^{(1)} + \left(3\Delta_2' - \partial_{T'}\right) w_z^{(3)} + \left(\mathrm{Ga} - 6\mathrm{La}\Delta_2\right) \Delta_2' \zeta^{(3)} \quad (56)$$

$$G_5^{(5)} = \partial_{T''} \Gamma^{(1)} + \left(\partial_{T'} - \mathrm{Sc}^{-1} \Delta_2'\right) \Gamma^{(3)}, \quad (57)$$

where C_{ij} are the convection terms given by

$$\begin{aligned}
\mathrm{C}_{ij} &= \nabla \times \nabla \times (\mathbf{u}^{(i)} \cdot \nabla \mathbf{u}^{(j)}) \cdot \mathbf{e}_3 \\
&= v_{zz} w_y + w_z v_{yz} + 2 v_y v_{yz} + v_z w_{yz} - 2 w_y w_{yz} \\
&+ w v_{yzz} + v_z v_{yy} - w_y v_{yy} - w_z w_{yy} - 2 v_y w_{yy} \\
&+ v v_{yyz} - w w_{yyz} - v w_{yyy} + 2 u_{yz} v_x + u_{zz} w_x \\
&- u_{yy} w_x + w_z u_{xz} + 2 u_x u_{xz} + 2 u_y v_{xz} + u_z w_{xz} \\
&- 2 w_x w_{xz} + w u_{xzz} + v_z u_{xz} + u_z v_{xz} - 2 u_y w_{xy} \\
&- 2 v_x w_{xz} + v u_{xyz} + u v_{xyz} - u w_{xyy} + u_z u_{xx} \\
&- w_x u_{xx} + w_y v_{xx} - w_z w_{xx} - 2 u_x w_{xx} + u u_{xxz} \\
&- w w_{xxz} - v w_{xxy} - u w_{xxx}
\end{aligned} \quad (58)$$

where the superscripts are suppressed and $v_{zz} w_y$ here denote $v_{zz}^{(i)} w_y^{(j)}$.

E. General solution and stationary pattern

To derive the general amplitude equation, we need to consider the stationary forms of the system variables $\Theta^{(m)} = (w^{(m)}, \zeta^{(m)}, \Gamma^{(m)})$. An ansatz is made of the initial condition of $\Theta^{(m)}$. Henceforth, to the ϵ^m-th order, we let

$$\Theta^{(m)} = A^{(m)}(\mathbf{x}_2', T', T'') \mathrm{e}^{\mathrm{i} m \omega T} \bar{\Theta}^{(m)} \Lambda^{(m)}(\mathbf{x}_2) + \text{c.c.}, \quad (59)$$

where c.c denote the complex conjugate and

$$\bar{\Theta}^{(m)} = (\bar{w}^{(m)}(z),\, \bar{\zeta}^{(m)}(z),\, \bar{\Gamma}^{(m)}), \quad (60)$$

$A(\mathbf{x}_2', T', T'')$ is the non-dimensional time and space-dependent amplitude, ω is the Floquet frequency and $\Lambda^{(m)}(\mathbf{x}_2)$ is the *stationary pattern function* in terms of the slow spatial variables $\mathbf{x}_2$; this denotes a quasi-stationary solution of the equation in the case of negligible driving force in the system. The realisation of the stationary pattern function allows the calculation of the coefficients of the amplitude equation in a closed form in the later section, as well as providing an ansatz for the initial condition of the surface at the onset of the pattern-forming instability. We note that the form of the stationary pattern function is idealised. We adapt the analytical solutions to the Swift-Hohenberg equations [31], which is the

governing equations for a soft wrinkling pattern forming process of a static soft sphere [9]. This introduces the assumption that the static pattern formation is a limiting case of the fluidic pattern formation and thus the adaptation of the initial condition is valid if the fluid motion does not deviate from quasi-staticity too far.

For planar fluid velocities $\mathbf{u}_2 = (u, v)$, we have

$$\mathbf{u}_2^{(m)} = A^{(m)}(\mathbf{x}_2', T', T'')\mathrm{e}^{im\omega T}(\bar{u}^{(m)}(z), \bar{v}^{(m)}(z))\Lambda^{(m)}(\mathbf{x}_2) + \text{c.c.} \tag{61}$$

To realise the regular hexagonal array in the first order of the asymptotic expansion, we consider the general function form

$$\Lambda_n^{(1)}(\mathbf{x}_2) = \sum_{i=1}^{n} \exp(\mathrm{i}\boldsymbol{\theta}_i^{(1)} \cdot \mathbf{x}_2), \tag{62}$$

where $n = 6$, $\mathbf{x}_2 = (x, y)$ and $\boldsymbol{\theta}_i^{(1)} = (\mathrm{Re}(\vartheta_i^{(1)}), \mathrm{Im}(\vartheta_i^{(1)}))$ for $\vartheta_i^{(1)}$ the sixth roots of unity forming a planar hexagon.

Extending to higher order, we construct $\vartheta_i^{(2)}$ to lie on the circumscribed circle of the previous hexagon such that, by Euclidean geometry, the radius of the successive circumscribed circle satisfy the geometric series

$$1, \frac{\sqrt{3}}{2}, \frac{3}{4}, \frac{3\sqrt{3}}{8}, \frac{9}{16}, \dots \tag{63}$$

Hence, to the m-th order, we have

$$\Lambda^{(m)}(\mathbf{x}_2; m, \mathbf{k}) \equiv \sum_{j} \Lambda_j^{(m)}(\mathbf{x}_2; m) \tag{64}$$

$$= \sum_{j=1}^{m}\sum_{i=1}^{n} \exp(\mathrm{i}\boldsymbol{\theta}_i^{(j)} \cdot \mathbf{x}_2) \tag{65}$$

where $\boldsymbol{\theta}_i^{(j)}$ for $j = 2, 3, 4$ are subsequent roots which lie on the hexagonal vertices. In explicit form, we have

$$2\Lambda_6^{(1)}(x, y) = \cos x + \cos\left[\tfrac{1}{2}(x + \sqrt{3}y)\right] + \cos\left[\tfrac{1}{2}(x - \sqrt{3}y)\right] \tag{66}$$

$$2\Lambda_6^{(2)}(x, y) = \cos 2x + \cos(x + \sqrt{3}y) + \cos(x - \sqrt{3}y) + \cos\sqrt{3}y + \cos\left[\tfrac{1}{2}(3x + \sqrt{3}y)\right] + \cos\left[\tfrac{1}{2}(3x - \sqrt{3}y)\right] \tag{67}$$

$$2\Lambda_6^{(3)}(x, y) = \cos 3x + \cos\left[\tfrac{3}{2}(x + \sqrt{3}y)\right] + \cos\left[\tfrac{3}{2}(x - \sqrt{3}y)\right] + \cos\tfrac{3}{2}\sqrt{3}y + \cos\left[\tfrac{3}{4}(3x + \sqrt{3}y)\right] + \cos\left[\tfrac{3}{4}(3x - \sqrt{3}y)\right] + \cos\tfrac{9}{4}x + \cos\left[\tfrac{9}{8}\left(x + \sqrt{3}y\right)\right] + \cos\left[\tfrac{9}{8}\left(-x + \sqrt{3}y\right)\right] \tag{68}$$

$$\vdots$$

This process is easily generalised to an arbitrary n-gon. Moreover, we evaluate the stationary pattern function $\Lambda(\mathbf{x}_2; m, k)$ at the origin $\mathbf{x}_2 = (0, 0)$ using the normalised hexagonal configuration such that

$$6\Lambda^{(1)}(x, y) = \mathrm{e}^{\mathrm{i}x} + \mathrm{e}^{\mathrm{i}(x+\sqrt{3}y)/2} + \mathrm{e}^{\mathrm{i}(x-\sqrt{3}y)/2} + \text{c.c} \tag{69}$$

$$12\Lambda^{(2)}(x, y) = \Lambda^{(1)}(2x, 2y) + \mathrm{e}^{3iy} + \mathrm{e}^{\mathrm{i}(3x+\sqrt{3}y)/2} + \mathrm{e}^{\mathrm{i}(-3x+\sqrt{3}y)/2} + \text{c.c} \tag{70}$$

$$18\Lambda^{(3)}(x, y) = \Lambda^{(1)}(3x, 3y) + \Lambda^{(2)}(\tfrac{3}{2}x, \tfrac{3}{2}y) + \mathrm{e}^{9\mathrm{i}x/4} + \mathrm{e}^{\frac{9}{8}\mathrm{i}(x+\sqrt{3}y)} + \mathrm{e}^{\frac{9}{8}\mathrm{i}(x-\sqrt{3}y)} + \text{c.c} \tag{71}$$

$$24\Lambda^{(4)}(x, y) = \Lambda^{(1)}(4x, 4y) + \Lambda^{(2)}(2x, 2y) + \Lambda^{(3)}(\tfrac{4}{3}x, \tfrac{4}{3}y) + \mathrm{e}^{3\sqrt{3}\mathrm{i}y/2} + \mathrm{e}^{\frac{9}{4}\mathrm{i}(x+\sqrt{3}y)} + \mathrm{e}^{\frac{9}{4}\mathrm{i}(-x+\sqrt{3}y)} + \text{c.c} \tag{72}$$

Hence we have

$$\Lambda^*\Lambda^{(1)} = 1 \tag{73}$$

$$\Lambda^*\Delta_2\Lambda^{(1)} = -\tfrac{1}{4}. \tag{74}$$

Similarly, $\Lambda^*\Lambda^{(m)} = 1$, we have

$$\Lambda^*\Delta_2\Lambda^{(2)} = -\tfrac{7}{8} \tag{75}$$

$$\Lambda^*\Delta_2\Lambda^{(3)} = -\tfrac{111}{64} \tag{76}$$

$$\Lambda^*\Delta_2\Lambda^{(4)} = -\tfrac{271}{64} \tag{77}$$

$$\vdots \tag{78}$$

to be used when obtaining the coefficients in the asymptotic expansion.

F. Solvability condition and the amplitude equation

We now consider the solvability condition of the asymptotic expansion and consequently, the derivation of the amplitude equation at various orders of the expansion parameter ϵ. Let $\langle \mathrm{f}, \mathrm{g}\rangle$ be the scalar product on $\Omega = \Omega_2 \times [-H, 0]$ by

$$\langle \mathrm{f}, \mathrm{g}\rangle = \int\int_{\Omega} \mathrm{fg}\, \mathrm{d}\Omega\, \mathrm{d}T, \tag{79}$$

let $\Theta^{(m)} = (w^{(m)}, h^{(m)}, \Gamma^{(m)})$ satisfy the $O(\epsilon^{m/2})$ problem $\mathcal{L}\Theta^{(m)} = G^{(m)}$ and $\Theta^* = (w^*, h^*, \Gamma^*)$ to satisfy the linear adjoint problem $\mathcal{L}^*\Theta^* = 0$.

By the Fredholm alternative theorem [42],the solvability condition to the $\epsilon^{(m+1)/2}$-th order is given by

$$\langle \Theta^*, G^{(m)}\rangle = 0. \tag{80}$$

For the tangential and normal interfacial stress conditions, we augment Θ^* with (w^*, w_z^*) and so the solvability

condition reads

$$0 = \int \left\{ \int_{\Omega} \left[\left(w^* G_1^{(m)} \right) \mathrm{d}\Omega + \int_{\Omega_2} h^* G_2^{(m)} \Big|_{z=0} \mathrm{d}\Omega_2 \right] \mathrm{d}T + \int_{\Omega_2} \left(w^* G_3^{(m)} + w_z^* G_4^{(m)} + \Gamma^* G_5^{(m)} \right) \Big|_{z=0} \mathrm{d}\Omega_2 \right\} \mathrm{d}T. \quad (81)$$

This solvability condition will yield [43] the amplitude equations across the asymptotic expansion scales and in what follows, we calculate the exact form of those amplitude equations in ascending order. A frequent notation used below is the angle and square bracket, where

$$\langle X^* X \rangle = \frac{1}{2} \int_{-1}^{1} X^* X dZ \quad (82)$$

$$[X^* X] = \frac{1}{2} X^* X \Big|_{-1}^{1}. \quad (83)$$

The angle bracket represents the integral of the quantities within across the area of the interface, typically used to calculate the velocity quantity, whereas the square brackets account for the jump in value of the quantity across the surface, often used for interfacial quantities such as thickness and surfactant concentration.

At $O(\epsilon^3)$, the amplitude equation is given by

$$a_1 \frac{\partial A}{\partial T'} + a_2 \Delta_2' A + a_3 A = 0, \quad (84)$$

where

$$a_1 = \Delta \langle w^* w^{(1)} \rangle + \langle \zeta^* \zeta^{(1)} \rangle - [w_z^* w_z^{(1)}] + [\Gamma^* \Gamma^{(1)}] \quad (85)$$

$$a_2 = (\partial_T - 6\Delta_2) \langle w^* w^{(1)} \rangle + [w^* w^{(1)}] + \mathrm{M}[w^* \Gamma^{(1)}] + 3[w_z^* w_z^{(1)}] + (\mathrm{Ga} - 6\mathrm{La}\Delta_2)[w_z^* \zeta^{(1)}] - \mathrm{Pe}^{-1}[\zeta^* \Gamma^{(1)}] + [\zeta^* u_2^{(1)} \zeta^{(1)}] \quad (86)$$

$$a_3 = -[\Gamma^* w_z^{(1)} \Gamma^{(1)}]. \quad (87)$$

and $u_2^{(i)} = u^{(i)} + v^{(i)}$.

At $O(\epsilon^4)$, we have

$$b_1 \frac{\partial A^2}{\partial T'} + b_2 \Delta_2' A + b_3 \Delta_2' A^2 + b_4 A + b_5 A^2 = 0, \quad (88)$$

where

$$b_1 = \Delta \langle w^* w^{(2)} \rangle + [\zeta^* \zeta^{(2)}] - [w_z^* w_z^{(2)}] + [\Gamma^* \Gamma^{(2)}] \quad (89)$$

$$b_2 = [\zeta^* u_2^{(2)} \zeta^{(1)}] \quad (90)$$

$$b_2 = (\partial_T - 6\Delta_2) \langle w^* w^{(2)} \rangle + [w^* w^{(2)}] + \mathrm{M}[w^* \Gamma^{(2)}] + 3[w_z^* w_z^{(2)}] + (\mathrm{Ga} - 6\mathrm{La}\Delta_2)[w_z^* \zeta^{(2)}] - \mathrm{Pe}^{-1}[\Gamma^* \Gamma^{(2)}] + [\zeta^* u_2^{(1)} \zeta^{(2)}] \quad (91)$$

$$b_3 = -[\Gamma^* w_z^{(2)} \Gamma^{(1)}] \quad (92)$$

$$b_4 = -[\Gamma^* w_z^{(1)} \Gamma^{(2)}]. \quad (93)$$

Similarly, at $O(\epsilon^5)$, we have

$$0 = c_1 \frac{\partial A}{\partial T''} + c_2 \frac{\partial \Delta_2' A}{\partial T'} + c_{31} \Delta_2' A + c_{32} \Delta_2' A^2 + c_{33} \Delta_2' A^3 + c_4 \frac{\partial A^3}{\partial T'} + c_5 \Delta_2^{2'} A + c_6 A^2 + c_7 A^3. \quad (94)$$

Consider that

$$\frac{\partial A^3}{\partial T'} = -3A^2 \left(\frac{a_2}{a_1} \Delta_2' A + \frac{a_3}{a_1} A \right) \quad (95)$$

where we can evaluate the nonlinear term using $A = \mathcal{A} \cos kx$, then

$$\int_{\Omega_2} \mathcal{A}^3 k^2 \cos kx \, dA \sim -\frac{2}{3} a_{21} k^2 |\Omega_2| A^3. \quad (96)$$

So the total contribution can be approximated by $-3a_{31} + 2a_{21}$ in the coefficient for the A^3 term.

For terms with spatial-temporal derivatives, apply the spatial operator to the lower-order equation, e.g. in this case, we apply Δ_2' to the $O(\epsilon^3)$ equation to yield

$$\frac{\partial \Delta_2' A}{\partial T'} = -\frac{a_2}{a_1} \Delta_2^{2'} A - \frac{a_3}{a_1} \Delta_2' A. \quad (97)$$

Whenceforth, after relabelling the coefficients, the amplitude equation at $O(\epsilon^5)$ is

$$0 = c_0 \frac{\partial A}{\partial T''} + \Delta_2' \left(c_1 A + c_2 A^2 + c_3 A^3 \right) + c_4 \Delta_2^{2'} A + c_5 A + c_6 A^2 + c_7 A^3 = 0, \quad (98)$$

where

$$c_0 = \Delta \langle w^* w^{(1)} \rangle - [\zeta^* \zeta^{(1)}] - [w_z^* w_z^{(1)}] + [\Gamma^* \Gamma^{(1)}] \quad (99)$$

$$c_1 = a_{31} \langle w^* w^{(1)} \rangle + [\zeta^* u_2^{(3)} \zeta^{(1)}] \quad (100)$$

$$c_2 = [\zeta^* u_2^{(2)} \zeta^{(2)}] \quad (101)$$

$$c_3 = (\partial_T - 6\Delta_2) \langle w^* w^{(3)} \rangle + [w^* w^{(3)}] + 3[w_z^* w_z^{(3)}] - \mathrm{Pe}^{-1}[\Gamma^* \Gamma^{(3)}] + (\mathrm{Ga} - 6\mathrm{La}\Delta_2)[w_z^* \zeta^{(3)}] + \mathrm{M}[w^* \Gamma^{(3)}] + [\zeta^* u_2^{(1)} \zeta^{(3)}] \quad (102)$$

$$c_4 = a_{21} \langle w^* w^{(1)} \rangle - \mathrm{La}[w_z^* \zeta^{(1)}] - \langle w^* w^{(1)} \rangle \quad (103)$$

$$c_5 = -[\Gamma^* w_z^{(3)} \Gamma^{(1)}] \quad (104)$$

$$c_6 = -[\Gamma^* w_z^{(2)} \Gamma^{(2)}] \quad (105)$$

$$c_7 = -[\Gamma^* w_z^{(1)} \Gamma^{(3)}] + (3a_{31} + 2a_{21}) \langle w^* w^{(3)} \rangle \quad (106)$$

for $a_{j1} = a_j / a_1$ for $j = 2, 3$.

G. Coefficient calculation under the thin-film assumption

In order to solve the linear system in equations (42-47) in a closed form, we substitute the general solution

(59) in the expanded asymptotic equations of motion, the left-hand side reduces to

$$\left[\mathrm{R}_m(k^2+\partial_z^2)-(k^4+\partial_z^4)\right]\bar{w}^{(m)}=0 \tag{107}$$

$$\left(k^2-\partial_z^2\right)\bar{w}^{(m)}\Big|_{z=\zeta}-\mathrm{M}\,k^2\Gamma^{(m)}=0 \tag{108}$$

$$\left(3k^2+\partial_z^2-\mathrm{R}_m\right)\bar{w}_z^{(m)}\Big|_{z=\pm\zeta} +(\mathrm{Ga}-\mathrm{La}\,k^2)k^2\bar{\zeta}^{(m)}=0 \tag{109}$$

$$R_m\Gamma^{(m)}-w_z|_{z=\pm\zeta}-\mathrm{Sc}^{-1}\Delta_2\Gamma^{(m)}=0 \tag{110}$$

$$\bar{w}^{(m)}\Big|_{z=\pm\zeta}=R_m\zeta \tag{111}$$

where $\mathrm{R}_m=\mathrm{i}m\omega$, for $\omega=\omega_1+\mathrm{i}\omega_2$.

Overdamped real solution. The condition that this linear system admits real solution necessarily imposes a condition on the frequency ω, i.e. we need ω to be purely complex and so $\omega_1=0$ and $\omega_2\in\mathbb{R}$. Therefore, this suggests that in order for the pattern-forming instability to be real and observable experimentally, we require the surface capillary wave to be in the overdamped regime, i.e. with wavelength $\lambda<\lambda_c$ where λ_c is the critical damping wavelength [29].

General solution. Supposing the system is in overall overdamped regime and $R_m\in\mathbb{R}$ as described in the previous subsection, the general solution to equation (107) is given by

$$\begin{aligned}\bar{w}^{(m)}=&\frac{k^2}{2}\left(\zeta^2-z^2\right)+\frac{k^3}{2m}\left(\zeta^2-z^2\right)+m\zeta k\\&+\frac{c_1}{mk}\left(\cos\sqrt{mk}\zeta-\cos\sqrt{mk}z\right)\\&+\frac{c_2}{mk}\left((z/\zeta)\sin\sqrt{mk}\zeta-\sin\sqrt{mk}z\right).\end{aligned} \tag{112}$$

By symmetry, $\bar{w}^{(m)}$ is an even function so $c_2=0$. The surfactant transport gives

$$\Gamma^{(m)}\sim\frac{\zeta}{k^2}\left(-c_1+k^2+\frac{k^3}{m}\right)\mathrm{Sc} \tag{113}$$

The tangential stress condition in equation (108) yields

$$c_1\sim\frac{k^3}{m}\left(1+\zeta m^2\right)+\frac{k^2}{m}(m+\mathrm{M}\Gamma^{(m)}) \tag{114}$$

Substituting the leading-order term for $\Gamma^{(m)}$ gives

$$c_1\sim\frac{k^3}{m}\left(1+\zeta m^2\right)+k^2+\frac{\zeta\mathrm{M}}{mk}\left(-c_1+k^2+\frac{k^3}{m}\right)\mathrm{Sc}. \tag{115}$$

To the leading order, we have

$$c_1\sim\frac{k^3}{m} \tag{116}$$

$$\Gamma^{(m)}\sim\frac{\zeta k}{m}\mathrm{Sc} \tag{117}$$

Taylor expansion gives

$$\cos\sqrt{mk}\zeta-\cos\sqrt{mk}z\sim\frac{1}{2}mk\left(z^2-\zeta^2\right)+\cdots \tag{118}$$

and therefore

$$\frac{c_1}{mk}\left(\cos\sqrt{mk}\zeta-\cos\sqrt{mk}z\right)=O\left(\frac{k^3\zeta^2}{m}\right). \tag{119}$$

The boundness condition on $\bar{w}^{(m)}$ then gives the criteria $\zeta^2k^3\sim 1$ and thus to leading-order, we have

$$\bar{w}^{(m)}\sim\frac{k^3}{2m}\left(\zeta^2-z^2\right) \tag{120}$$

where, since the wavenumber and the wavelength λ are inversely proportional, we conclude that the amplitude ζ and the wavelength of the instability λ are related by the power-law relation

$$\bar{\lambda}\sim\bar{\zeta}^{2/3}. \tag{121}$$

where $\bar{\lambda}=\lambda/\lambda_0$ and $\bar{\zeta}=\zeta/\zeta_0$ are non-dimensional wavelength and interface height, and λ_0 and ζ_0 are their reference values, respectively. This is an important relation since ζ can be experimentally measured and thus by extension, this relation allows for the wavelength of the instability to be measured as well.

Non-dimensionalising $\bar{w}$ gives

$$\bar{W}^{(m)}\sim\frac{1-Z^2}{2m} \tag{122}$$

where $W=w/w_0$ and $Z=z/\zeta$, w_0 the reference velocity. Similarly, we consider the non-dimensionless length $(X,Y)=k(x,y)$ and that $(\Gamma,\zeta)\to(\Gamma/\Gamma_0,\zeta/\zeta_0)$ for Γ_0 the reference surfactant concentration.

Supposing we wish to obtain $\Gamma^{(m)}$ in terms of M, consider the non-dimensional tangential condition

$$\left(1-\partial_{ZZ}\right)\bar{W}^{(m)}\Big|_{Z=1}-\mathrm{M}\Gamma^{(m)}=0 \tag{123}$$

and so we have

$$\Gamma^{(m)}\sim\frac{1}{m}\mathrm{M}^{-1}. \tag{124}$$

Similarly, to obtain $\zeta^{(m)}$ in terms of m, we use the normal condition that

$$-\frac{(3k^2-R_mk_0^2)}{(\mathrm{Ga}k_0^2-\mathrm{La}k^2)}\frac{1}{m}=\zeta^{(m)}. \tag{125}$$

To the leading order, $\zeta^{(m)}$ is given by

$$\zeta^{(m)}\sim\frac{3}{m}\mathrm{La}^{-1}. \tag{126}$$

Additional remarks. We have two asymptotic scaling for $\Gamma^{(m)}$ arising naturally from the calculations in this

section. One candidate scaling comes from the surfactant transport and the normal stress balance, which yields $\Gamma^{(m)} \sim 3\,\mathrm{Sc}\,\mathrm{La}^{-1}k/m^2$, in terms of the Schmidt and the Laplace number. The second candidate comes from the tangential stress balance term given by $\Gamma^{(m)} \sim \mathrm{M}^{-1}/m$, in terms of the Marangoni number. To keep the theory as simple as possible, we choose the latter scaling due to the dominance of Marangoni convection over Marangoni diffusion for majority of the lengthscales present in this study. For very thin films where $\lambda \sim \lambda_c^{(0)}$, where $\lambda_c^{(0)}$ is the critical damping wavelength in absence of surfactants, it follows that the Schmidt number asymptotic scaling be used.

Calculation of coefficients. Finally, having found the asymptotic scalings for the various system variables, we recall the non-dimensional form of the expansion brackets

$$\langle X^* X\rangle = \frac{1}{2}\int_{-1}^{1} X^* X dZ \tag{127}$$

$$[X^* X] = \frac{1}{2} X^* X\Big|_{-1}^{1}. \tag{128}$$

With the substitution of the system variables into the bracket terms, we have

$$\langle \bar{W}^* \bar{W}^{(m)}\rangle = \frac{1}{2}\int_{-1}^{1} \frac{(1-\bar{Z}^2)(1-Z^2)}{4m} dZ \tag{129}$$

$$= \frac{2}{15m} \tag{130}$$

$$\langle \bar{W}^* \bar{W}^{(m)}_{ZZ}\rangle = \frac{1}{2}\int_{-1}^{1} \frac{\bar{Z}^2-1}{2m} dZ \tag{131}$$

$$= -\frac{2}{3m} \tag{132}$$

$$[\bar{W}^*_Z \bar{W}^{(m)}_Z] = \frac{|Z|^2}{2m}\Big|_{-1}^{1} \tag{133}$$

$$= 0 \tag{134}$$

$$[\zeta^* U_2^{(n)} \zeta^{(m)}] = \frac{9Z}{2mn}\Big|_{-1}^{1} \mathrm{La}^{-2} \tag{135}$$

$$= -\frac{9}{mn}\mathrm{La}^{-2} \tag{136}$$

$$[\Gamma^* W_Z^{(n)} \Gamma^{(m)}] = -\frac{Z}{2mn}\Big|_{-1}^{1} \mathrm{M}^{-2} \tag{137}$$

$$= -\frac{1}{mn}\mathrm{M}^{-2} \tag{138}$$

where the following terms vanish under the limit $k \gg 1$,

$$[W^* W^{(m)}], [\zeta^* \zeta^{(m)}], [\Gamma^* \Gamma^{(m)}], [W^* \Gamma^{(m)}], [W_Z^* \zeta^{(m)}]. \tag{139}$$

Finally, we can calculate the closed form of the coefficients of the amplitude equations. To proceed, we substitute the expansion terms in equations (130) to (138) into equation (98). Starting with $O(\epsilon^3)$, the leading-order terms are (in units of $a_1 = c_0 = -\frac{26}{15}$)

$$a_1 = \Delta\langle W^* W^{(1)}\rangle \tag{140}$$

$$a_2 = (\partial_T - 6\Delta_2)\langle w^* w^{(1)}\rangle + 3[w_z^* w_z^{(1)}] + [\zeta^* u_2^{(1)} \zeta^{(1)}] \tag{141}$$

$$a_3 = -[\Gamma^* w_z^{(1)} \Gamma^{(1)}], \tag{142}$$

similarly, we have

$$a_3 = -\mathrm{M}^{-2} \tag{143}$$

$$a_2 = \tfrac{8}{3} - 9\mathrm{La}^{-2} \tag{144}$$

To order $O(\epsilon^5)$, the coefficients are given by

$$c_0 = (\Delta_2 + \partial_{zz})\langle w^* w^{(1)}\rangle \tag{145}$$

$$= -\tfrac{19}{15} \tag{146}$$

$$c_1 = -a_{31}\langle w^* w^{(1)}\rangle + [\zeta^* u_2^{(3)} \zeta^{(1)}] \tag{147}$$

$$= \tfrac{2}{15}\mathrm{M}^{-2} - 3\mathrm{La}^{-1} \tag{148}$$

$$c_2 = [\zeta^* u_2^{(2)} \zeta^{(2)}] \tag{149}$$

$$= -\tfrac{9}{4}\mathrm{La}^{-2} \tag{150}$$

$$c_3 = -6\Delta_2\langle w^* w^{(3)}\rangle + [\zeta^* u_2^{(1)} \zeta^{(3)}] \tag{151}$$

$$= \tfrac{37}{80} - 3\mathrm{La}^{-2} \tag{152}$$

$$c_4 = (-a_{21} - 1)\langle w^* w^{(1)}\rangle \tag{153}$$

$$= -\tfrac{22}{45} + \tfrac{18}{15}\mathrm{La}^{-2} \tag{154}$$

$$c_5 = -[\Gamma^* w_z^{(3)} \Gamma^{(1)}] \tag{155}$$

$$= \tfrac{1}{3}\mathrm{M}^{-2} \tag{156}$$

$$c_6 = -[\Gamma^* w_z^{(2)} \Gamma^{(2)}] \tag{157}$$

$$= \tfrac{1}{4}\mathrm{M}^{-2} \tag{158}$$

and

$$c_7 = -[\Gamma^* w_z^{(1)} \Gamma^{(3)}] + (-3a_{31} + 2a_{21})\langle w^* w^{(3)}\rangle \tag{159}$$

$$= \tfrac{19}{45}\mathrm{M}^{-2} + \tfrac{6}{5}\mathrm{La}^{-2} - \tfrac{16}{45} \tag{160}$$

H. Stability diagrams and hysteresis cycles

Under the coefficients obtained under the overdamped capillary wave state in the previous subsections, we can summarise the dynamics of the system into regimes depending on the values of the Laplace and the (velocity-independent) Marangoni numbers, La and M.

To recast the equation in the Cahn-Hilliard form, we require the coefficients of the Laplacian terms to satisfy the double-well potential, i.e. we need

$$c_1 = -\tfrac{1}{3}c_2 = \tfrac{1}{2}c_3. \tag{161}$$

The condition in equation (161) gives

$$\tfrac{2}{15}\mathrm{M}^{-2} - \mathrm{La}^{-2} = \tfrac{1}{4}\mathrm{La}^{-2} = \tfrac{117}{160} - \tfrac{1}{2}\mathrm{La}^{-2} \tag{162}$$

which yields the solution

$$\mathrm{La} = \left(\tfrac{40}{39}\right)^{1/2} \simeq 1.0127 \tag{163}$$

$$\mathrm{M} = \tfrac{8}{3\sqrt{65}} \simeq 0.3308. \tag{164}$$

To recast the amplitude equation in a normal form, we consider the scaling that

$$\tau = \frac{4c_4}{c_1^2}T'' \tag{165}$$

$$\Delta_X = k_c^{-2}\Delta_2' \tag{166}$$

$$\mathrm{A} = A\left(\frac{c_1^2}{4c_4c_7}\right)^{-1/2} \tag{167}$$

$$\alpha = \frac{4c_4c_5}{c_1^2} \tag{168}$$

$$\beta = \frac{2c_6}{c_1\sqrt{|c_7|/c_4}} \tag{169}$$

where $k_c^2 = |c_1|/2c_3$. Substituting back into the amplitude equation yields the normal form

$$\mathrm{A}_\tau = -\Delta\frac{\mathrm{d}f(\mathrm{A})}{\mathrm{dA}} + \Delta^2\mathrm{A} - \alpha\mathrm{A} - \beta\mathrm{A}^2 - \mathrm{A}^3, \tag{170}$$

where

$$f(\mathrm{A}) = \tfrac{1}{2}\mathrm{A}^2(1-\mathrm{A})^2 \tag{171}$$

represents a double-well energy potential.

Comparing with the stability calculations of the canonical form [9, 31], we have the different wrinkling phases which emerge depending on the parameters α and β in the following way:

$$\text{Hexagonal dots:} -\tfrac{1}{15}\beta^2 < 1-\alpha < \tfrac{4}{3}\beta^2 \tag{172}$$

$$\text{Mixed dots/Labyrinth:} \quad \tfrac{4}{3}\beta^2 < 1-\alpha < \tfrac{16}{3}\beta^2 \tag{173}$$

$$\text{Labyrinth:} \quad \tfrac{16}{3}\beta^2 < 1-\alpha \tag{174}$$

where

$$\alpha = \frac{\tfrac{4}{3}\mathrm{M}^{-2}\left(-\tfrac{38}{75} + \tfrac{6}{15}\mathrm{La}^{-2}\right)}{\left(\tfrac{2}{15}\mathrm{M}^{-2} - \mathrm{La}^{-2}\right)^2}, \tag{175}$$

$$\beta = \frac{\mathrm{M}^{-2}}{\tfrac{2}{15}\mathrm{M}^{-2} - \mathrm{La}^{-2}}\sqrt{\frac{-\tfrac{38}{75} + \tfrac{6}{15}\mathrm{La}^{-2}}{\tfrac{13}{15}\mathrm{M}^{-2} + \tfrac{4}{15}\mathrm{La}^{-2} + \tfrac{56}{225}}}. \tag{176}$$

In addition, the stability criterion in the SH-regime predicts the existence of two hysteresis cycles similar to the existing literature [9, 44, 45]. To sketch the hysteresis curves, the first hysteresis cycle occurs near the transcritical bifurcation point $1-\alpha = 0$ and encompasses the subcritical hexagaonal (hysteresis) region $-\frac{1}{15}\beta^2 < 1-\alpha < 0$. Within the subcritical hysteresis region, we have the possible stable co-existence of both the hexagonal dot state and the no-pattern state. For the labyrinth state, a consideration of the stationary solution of the SH equation [31] sets up the second hysteresis cycle in the range $\frac{4}{3}\beta^2 < 1-\alpha < \frac{4}{16}\beta^2$.

I. Finite element simulations

To simulate the amplitude equation in equation (170) is not a trivial task. While the double-well potential term is reminiscent of the Cahn-Hilliard equation, the polynomial terms are a feature of the Swift-Hohenberg equation, coupled with a fourth-order bi-Laplacian operator, this presents challenges within the finite element method. To employ a Galerkin-method requires either a piecewise smooth and globally C^1-continuous element for a basis function or a mixed formulation which bypasses the C^1-continuous requirement by introducing an auxiliary field to recast the fourth-order equation of motion into two coupled second-order equations. In this work, we shall use the mixed formulation which has shown for Cahn-Hilliard equations [46] to be less computationally expensive with a comparable accuracy than C^1-continuous methods. We outline below how the two finite-element methods differ in their weak-form formulations.

Let $(\cdot,\cdot)$ and $\langle\cdot,\cdot\rangle$ denote interior and boundary inner products, respectively, the amplitude equation (170) can be written as

$$\frac{\partial\mathrm{A}}{\partial t} = \Delta\left[M\left(\frac{\mathrm{d}f}{\mathrm{dA}} - \varepsilon\Delta\mathrm{A}\right)\right] + g(\mathrm{A}), \tag{177}$$

where M is a mobility of A, $f(\mathrm{A})$ is a free energy density and $\varepsilon \in \mathbb{R}$. Let $\mathcal{H}^2$ be the Hilbert space of functions with square-integrable second derivatives and multiply equation (177) by a test function $\phi \in \mathcal{H}^2$ and satisfying the boundary conditions; we find $\mathrm{A} \in \mathcal{H}^2$ where

$$\begin{aligned}\left(\frac{\partial\mathrm{A}}{\partial t} - g(\mathrm{A}), \phi\right) &= -\varepsilon\left(\Delta\mathrm{A}, \nabla\cdot(M\nabla\phi)\right)\\ &\quad - \left(M\nabla\frac{\mathrm{d}f}{\mathrm{dA}}, \nabla\phi\right)\\ &\quad + \left\langle M\frac{\partial}{\partial n}\left(\frac{\mathrm{d}f}{\mathrm{dA}} - \varepsilon\Delta\mathrm{A}\right), \phi\right\rangle\\ &\quad + \left\langle \varepsilon\Delta\mathrm{A}, M\frac{\partial\phi}{\partial n}\right\rangle,\end{aligned} \tag{178}$$

where $\partial/\partial n = \nabla\cdot\mathbf{n}$ is the normal derivative. This approach requires the use of C^1-continuous elements in the Laplacian term. To circumvent this requirement and to continue to use C^0-continuous elements, we need to split this term into two expressions and the amplitude

equation (170) can be written [47] as

$$\frac{\partial \mathrm{A}}{\partial t} = \nabla \cdot (M \nabla c) + g(\mathrm{A}), \tag{179}$$

$$c = \frac{\mathrm{d}f}{\mathrm{dA}} - \varepsilon \Delta \mathrm{A}, \tag{180}$$

where c is known as the chemical potential. This is known as the mixed formulation. To find $(\mathrm{A}, c) \in \mathcal{H}^1 \times \mathcal{H}^1$ satisfying the boundary conditions, we consider the weak form

$$\left(\frac{\partial \mathrm{A}}{\partial t} - g(\mathrm{A}), \phi\right) = -\left(M \nabla c, \nabla \phi\right) + \left\langle M \frac{\partial c}{\partial n}, \phi \right\rangle \tag{181}$$

$$(c, \psi) = \left(\frac{\mathrm{d}f}{\mathrm{dA}}, \psi\right) + \varepsilon \left(\nabla \mathrm{A}, \nabla \psi\right) - \varepsilon \left\langle \frac{\partial \mathrm{A}}{\partial n}, \psi \right\rangle \tag{182}$$

for auxiliary test function $\psi \in \mathcal{H}$.

Linear and quadratic Lagrange finite elements are used to discretise equations (181) and (182) in 2-d space. The mesh used for the Galerkin projection is a triangulation of the interface with up to 97,000 elements for the concave case and 87,000 elements for the convex case. The FEM package FENiCS [38] is used for the calculation, whereby any integrals over the manifold mesh can be reduced to integrals over the surface facets of a mesh [39].

The Crank-Nicolson method is used for the discretisation in time and the Newton-Krylov solvers based on PETSc's SNES module is used with the discretisations in space and time solved using the generalised minimal residual method (GMRES). Each iteration is solved to a relative tolerance of 10^{-6}. The simulations were carried out on a laptop PC with 4 cores at 2.5GHz. The typical computational time for each of the subfigures in Fig. 4-5 of the main paper were 5-8 hours. We ran the same simulation on another computer with 16 cores at 2.8GHz, the runtime reduced to about 3 hours. Hence the solution can be scaled even further if more computational resources are available. The parallel MPI routine used is a built-in feature of the FENiCs finite element package.

J. Coupling with thin-film flow

In regions where the pattern instability is not triggered, the bubble membrane undergoes drainage flow under the influence of gravity and Marangoni effect. Incorporating the Marangoni effect, we have the system of equation [48, 49]

$$h_t + \nabla \cdot \left(\tfrac{1}{2} h^2 \nabla \Gamma + \tfrac{1}{3} h^3 \left(\mathrm{C}\Delta - \mathrm{Bo}\right) \nabla h\right) = 0 \tag{183}$$

$$\Gamma_t + \nabla \cdot \left(\tfrac{1}{2} \Gamma h^2 \left(\mathrm{C}\Delta - \mathrm{Bo}\right) \nabla h - \Gamma h \nabla \Gamma\right) = \mathrm{Pe}^{-1} \Delta \Gamma \tag{184}$$

where h_0 is a reference film thickness, $\mathrm{Bo} = \rho g h_0/\Delta\sigma$, $\mathrm{C} = \bar{\epsilon}^2 \sigma_{\max}/\Delta\sigma$ and $\mathrm{Pe} = (\Delta\sigma) h_0/\mu D_s$ are the Bond, capillary and Péclet numbers, respectively, where $\Delta\sigma = \sigma_0 - \sigma_{\max}$ is the difference in surface tension coefficient, $\sigma_0, \sigma_{\max}$ are the initial and maximum surface tension coefficient, D_s is the surface diffusivity of the surfactant and $\bar{\epsilon} = h_0/L \ll 1$ is the lubrication condition. We note the appearance of the Péclet number as opposed to the Schmidt number as in the equations of motion in the pattern formation. This is due to the increasing relevance of convectional forces compared with diffusion outside of the instability region and so a fluid velocity-independent formulation is no longer required in this region.

For the simulations in Supplementary Video 4, we neglect the right-hand-side diffusion term in equation (184) by considering [29] a wavelength $\lambda > 4\lambda_c^{(0)}$, where $\lambda_c^{(0)}$ is the critical damping wavelength. Integrating equations (183) and (184) gives the weak form

$$(\partial_t h, \phi) = \left(\tfrac{1}{2} h^2 \nabla \Gamma - \tfrac{1}{3} h^3 \mathrm{Bo} \nabla h, \nabla \phi\right) + \tfrac{1}{3}\left(\mathrm{C} h^3 \nabla c, \nabla \phi\right) - \tfrac{1}{3}\left\langle \mathrm{C} h^3 \partial_n c, \phi\right\rangle \tag{185}$$

$$(c, \psi) = (\nabla h, \nabla \psi) - \langle \partial_n h, \psi \rangle \tag{186}$$

$$(\partial_t \Gamma, \varphi) = \left(-\Gamma h \nabla \Gamma - \tfrac{1}{3} \Gamma h^2 \mathrm{Bo} \nabla h, \nabla \varphi\right) + \tfrac{1}{2}\left(\mathrm{C} \Gamma h^2 \nabla d, \nabla \varphi\right) - \tfrac{1}{2}\left\langle \mathrm{C} \Gamma h^2 \partial_n d, \varphi\right\rangle \tag{187}$$

$$(d, \vartheta) = (\nabla h, \nabla \vartheta) - \langle \partial_n h, \vartheta \rangle \tag{188}$$

where $\phi, \psi, \varphi, \vartheta \in \mathcal{H}^2$ are test functions and $c, d \in \mathcal{H}^1$ are auxiliary functions. The equations are then evolved across the entire hemisphere in the same manner as the amplitude equations in the previous section using the same mesh. We note that these terms are of order $O(h^2)$ and $O(h^3)$ and thus would be negligible if $h \to 0$, the region where the instability dominates over the thin-film flow. Outside of this region where $h = O(1)$, the pattern formation equations are negligible and the thin-film equations dominate the overall dynamics.

II. OPTICS OF THE EXPERIMENT

In terms of optics of the system, with schematic of the setup shown in figure (3a), light strikes the thin-film membrane, with refractive index $n_{\mathrm{film}} > 1$, after it travels through the air with refractive index $n_{\mathrm{air}} = 1$. The reflection at the upper air-film boundary introduces a phase-shift with phase π in the reflected wave since $n_{\mathrm{air}} < n_{\mathrm{film}}$. At the bottom film-air boundary the reflection will not change the phase of the reflected wave because $n_{\mathrm{film}} > n_{\mathrm{air}}$. The constructive and destructive interference of reflected light is then given by

$$2 n_{\mathrm{film}} h \cos\theta_2 = \begin{cases} \left(m - \frac{1}{2}\right)\lambda & \text{constructive interference} \\ m\lambda & \text{destructive interference} \end{cases} \tag{189}$$

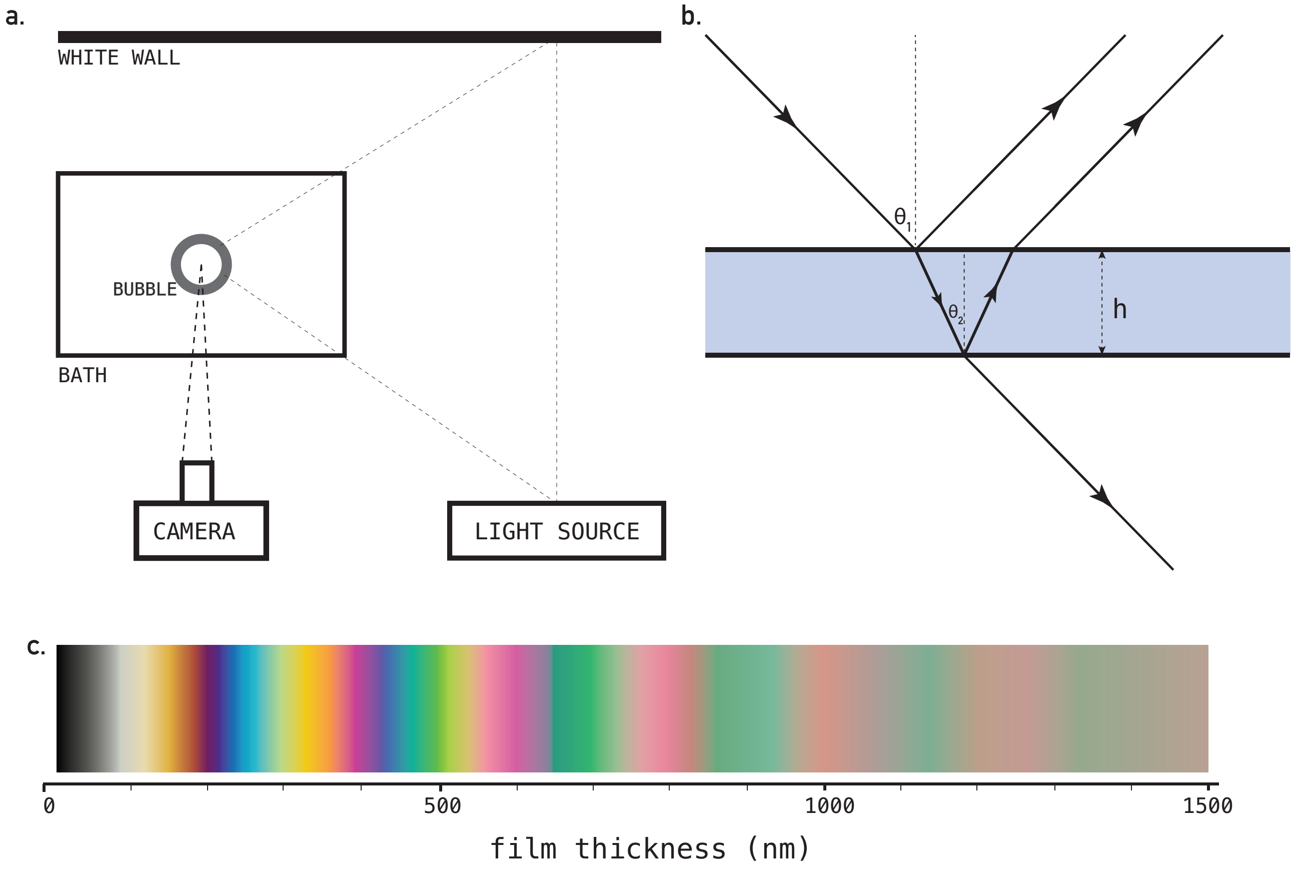

S3. **Schematics of interferometry.** a. Schematic of experimental setup of concave bubble membrane interferometry, b. schematic of the interferometry process and c. colour spectrum relating to film thickness, calculated using interferometry.

where h is the film thickness, n_{film} is the refractive index of the thin-film, θ_2 is the angle of incidence of the wave on lower film-air boundary, λ is the wavelength of the light and $m \in \mathbb{N}$. This is summarised in figure (3b).

We can then determine the wavelength-colour correspondence with figure (3c). Note here that the left-most portion of figure (3c), i.e. the region under 380nm is an extrapolation based on the data of the visible spectrum, which resides in the region of approximately 380 to 650nm.

REFERENCES AND NOTES

1. P. W. Higgs, Broken symmetries and the masses of gauge bosons. *Phys. Rev. Lett.* **13**, 508–509 (1964).

2. A. Onuki, *Phase Transition Dynamics* (Cambridge Univ. Press, 2002).

3. A. R. Palmer, Symmetry breaking and the evolution of development. *Science* **306**, 828–833 (2004).

4. V. L. Ginzburg, L. D. Landau, On the theory of superconductivity. *J. Exp. Theor. Phys.* **20**, 1064–1082 (1950).

5. J. W. Cahn, J. E. Hilliard, Free energy of a nonuniform system. I. Interfacial free energy. *J. Chem. Phys.* **28**, 258–267 (1958).

6. J. Swift, P.C. Hohenberg, Hydrodynamic fluctuations at the convective instability. *Phys. Rev. A* **15**, 319 (1977).

7. A. M. Turing, The chemical basis of morphogenesis. *Bull. Math. Biol.* **52**, 153–197 (1990).

8. J. E. Marsden, L. Sirovich, S. S. Antman, G. Iooss, P. Holmes, D. Barkley, M. Dellnitz, P. Newton, in *Introduction to Applied Nonlinear Dynamical Systems and Chaos*, S. Wiggins, Ed. (Springer, 2003).

9. N. Stoop, R. Lagrange, D. Terwagne, P. M. Reis, J. Dunkel, Curvature-induced symmetry breaking determines elastic surface patterns. *Nat. Mater.* **14**, 337–342 (2015).

10. T. Tallinen, J. Y. Chung, F. Rousseau, N. Girard, J. Lefèvre, L. Mahadevan, On the growth and form of cortical convolutions. *Nat. Phys.* **12**, 588–593 (2016).

11. E. Cerda, L. Mahadevan, Geometry and physics of wrinkling. *Phys. Rev. Lett.* **90**, 074302 (2003).

12. K. Efimenko, M. Rackaitis, E. Manias, A. Vaziri, L. Mahadevan, J. Genzer, Nested self-similar wrinkling patterns in skins. *Nat. Mater.* **4**, 293–297 (2005).

13. G. Cao, X. Chen, C. Li, A. Ji, Z. Cao, Self-assembled triangular and labyrinth buckling patterns of thin films on spherical substrates. *Phys. Rev. Lett.* **100**, 036102 (2008).

14. N. Bowden, S. Brittain, A. G. Evans, J. W. Hutchinson, G. M. Whitesides, Spontaneous formation of ordered structures in thin films of metals supported on an elastomeric polymer. *Nature* **393**, 146–149 (1998).

15. E. P. Chan, A. J. Crosby, Fabricating microlens arrays by surface wrinkling. *Adv. Mater.* **18**, 3238–3242 (2006).

16. P. D. Howell, Surface-tension-driven flow on a moving curved surface. *J. Eng. Math.* **45**, 283–308 (2003).

17. V. G. Levich, V. S. Krylov, Surface-tension-driven phenomena. *Annu. Rev. Fluid Mech.* **1**, 293–316 (1969).

18. A. Oron, S. H. Davis, S. G. Bankoff, Long-scale evolution of thin liquid films. *Rev. Mod. Phys.* **69**, 931–980 (1997).

19. J. U. Brackbill, D. B. Kothe, C. Zemach, A continuum method for modeling surface tension. *J. Comput. Phys.* **100**, 335–354 (1992).

20. F. Denner, F. Evrard, R. Serfaty, B. G. M. van Wachem, Artificial viscosity model to mitigate numerical artefacts at fluid interfaces with surface tension. *Comput. Fluids* **143**, 59–72 (2017).

21. L. Shen, F. Denner, N. Morgan, B. van Wachem, D. Dini, Before the bubble ruptures. *Phys. Rev. Fluids* **2**, 090505 (2017).

22. R. V. Craster, O. K. Matar, Dynamics and stability of thin liquid films. *Rev. Mod. Phys.* **81**, 1131–1198 (2009).

23. B. K. P. Horn, B. G. Schunck, Determining optical flow. *Artif. Intell.* **17**, 185–203 1981.

24. B. D. Lucas, T. Kanade, An iterative image registration technique with an application to stereo vision, in *Proceedings of the 7th International Joint Conference on Artificial Intelligence* (Morgan Kaufmann Publishers Inc., 1981), vol. 2, pp. 674–679.

25. G. Farnebäck, Two-frame motion estimation based on polynomial expansion. *Scandinav. Conf. Image Analysis* **2749**, 363–370 (2003).

26. M. C. Cross, P. C. Hohenberg, Pattern formation outside of equilibrium. *Rev. Mod. Phys.* **65**, 851–1112 (1993).

27. V. V. Yaminsky, S. Ohnishi, E. A. Vogler, R. G. Horn, Stability of aqueous films between bubbles. Part 1. The effect of speed on bubble coalescence in purified water and simple electrolyte solutions. *Langmuir* **26**, 8061–8074 (2010).

28. F. Denner, Frequency dispersion of small-amplitude capillary waves in viscous fluids. *Phys. Rev. E* **94**, 023110 (2016).

29. L. Shen, F. Denner, N. Morgan, B. van Wachem, D. Dini, Capillary waves with surface viscosity. *J. Fluid Mech.* **847**, 644–663 (2018).

30. P. Pfeiffer, Q. Zeng, B. H. Tan, C.-H. Ohl, Merging of soap bubbles and why surfactant matters. *Appl. Phys. Lett.* **116**, 103702 (2020).

31. A. A. Golovin, A. A. Nepomnyashchy, *Self-Assembly, Pattern Formation and Growth Phenomena in Nano-Systems* (Springer, 2006).

32. D. Barbará, P. Chen, Using self-similarity to cluster large data sets. *Data Mining Knowl. Discov.* **7**, 123–152 (2003).

33. P. Pfeifer, Fractal dimension as working tool for surface-roughness problems. *Appl. Surf. Sci.* **18**, 146–164 (1984).

34. B. B. Chaudhuri, N. Sarkar, Texture segmentation using fractal dimension. *IEEE Trans. Pattern Anal. Mach. Intell.* **17**, 72–77 (1995).

35. D. Exerowa, A. Nikolov, M. Zacharieva, Common black and Newton film formation. *J. Colloid Interface Sci.* **81**, 419–429 (1981).

36. B. V. Derjaguin, A.V. Prokhorov, On the theory of the rupture of black films. *Prog. Surf. Sci.* **45**, 21–28 (1994).

37. L. Zhang, M. R. Tonks, D. Gaston, J. W. Peterson, D. Andrs, P. C. Millett, B. S. Biner, A quantitative comparison between C^0 and C^1 elements for solving the Cahn-Hilliard equation. *J. Comput. Phys.* **236**, 74–80 (2013).

38. M. S. Alnæs, J. Blechta, J. Hake, A. Johansson, B. Kehlet, A. Logg, C. Richardson, J. Ring, M. E. Rognes, G. N. Wells, The FEniCS Project Version 1.5. *Arch. Numer. Softw.* **3**, 9–23 (2015).

39. M. E. Rognes, D. A. Ham, C. J. Cotter, A. T. T. McRae, Automating the solution of PDEs on the sphere and other manifolds in FEniCS 1.2. *Geosci. Model Dev.* **6**, 2099–2119 (2013).

40. K. F. Riley, M. P. Hobson, S. J. Bence, *Mathematical Methods for Physics and Engineering* (Cambridge Univ. Press, ed. 3, 2004).

41. H. A. Stone, A simple derivation of the time-dependent convective-diffusion equation for surfactant transport along a deforming interface. *Phys. Fluids A* **2**, 111–112 (1990).

42. R. Courant, D. Hilbert, *Methods of Mathematical Physics* (Wiley, 1989).

43. A. C. Skeldon, G. Guidoboni, Pattern selection for Faraday Waves in an incompressible viscous fluid. *SIAM J. Appl. Math.* **67**, 1064–1100 (2007).

44. E. Pampaloni, C. Pérez-García, L. Albavetti, S. Ciliberto, Transition from hexagons to rolls in convection in fluids under non-Boussinesq conditions. *J. Fluid Mech.* **234**, 393–416 (1992).

45. C. Kubstrup, H. Herrero, C. Pérez-García, Fronts between hexagons and squares in a generalized Swift-Hohenberg equation. *Phys. Rev. E* **54**, 1560–1569 (1996).

46. S. Kaessmair, P. Steinmann, Comparative computational analysis of the Cahn-Hilliard equation with emphasis on C^1-continuous methods. *J. Comput. Phys.* **322**, 783–803 (2016).

47. C. M. Elliott, D. A. French, F. A. Milner, A second-order splitting method for the Cahn-Hilliard equation. *Numer. Math.* **54**, 575–590 (1989).

48. O. E. Jensen, J. B. Grotberg, Insoluble surfactant spreading on a thin viscous film: Shock evolution and film rupture. *J. Fluid Mech.* **240**, 259–288 (1992).

49. B. D. Edmonstone, O. K. Matar, R. V. Craster, Flow of surfactant-laden thin films down an inclined plane. *J. Eng. Math.* **50**, 141–156 (2004).
